# A computational and structural analysis of germline and somatic variants affecting the DDR mechanism, and their impact on human diseases

**DOI:** 10.1038/s41598-021-93715-6

**Published:** 2021-07-12

**Authors:** Lorena Magraner-Pardo, Roman A. Laskowski, Tirso Pons, Janet M. Thornton

**Affiliations:** 1 Prostate Cancer Clinical Unit, Spanish National Cancer Research Center (CNIO), Madrid, Spain; 2 European Molecular Biology Laboratory, European Bioinformatics Institute (EMBL-EBI), Cambridge, UK; 3 Department of Immunology and Oncology, National Center for Biotechnology, Spanish National Research Council (CNB-CSIC), Madrid, Spain

**Keywords:** Cancer, Computational biology and bioinformatics

## Abstract

DNA-Damage Response (DDR) proteins are crucial for maintaining the integrity of the genome by identifying and repairing errors in DNA. Variants affecting their function can have severe consequences since failure to repair damaged DNA can result in cells turning cancerous. Here, we compare germline and somatic variants in DDR genes, specifically looking at their locations in the corresponding three-dimensional (3D) structures, Pfam domains, and protein–protein interaction interfaces. We show that somatic variants in metastatic cases are more likely to be found in Pfam domains and protein interaction interfaces than are pathogenic germline variants or variants of unknown significance (VUS). We also show that there are hotspots in the structures of ATM and BRCA2 proteins where pathogenic germline, and recurrent somatic variants from primary and metastatic tumours, cluster together in 3D. Moreover, in the *ATM, BRCA1* and *BRCA2* genes from prostate cancer patients, the distributions of germline benign, pathogenic, VUS, and recurrent somatic variants differ across Pfam domains. Together, these results provide a better characterisation of the most recurrent affected regions in DDRs and could help in the understanding of individual susceptibility to tumour development.

## Introduction

DNA is subject to continuous damage-causing alterations in both somatic and germline tissues. Cells rely on their DNA-Damage Response (DDR) systems to repair the damage before it leads to serious harm, such as the cell turning cancerous. Different pathways are involved, depending on whether the DNA sequence damage is a mutated base, a single-strand break (SSB), or a double-strand break (DSB). These pathways include: Base Excision Repair (BER), Nucleotide Excision Repair (NER), Mismatch Repair (MMR), Homologous Recombination (HR) and Non-Homologous End Joining (NHEJ). Each pathway has its own set of DNA-repair proteins, encoded by the DDR genes^1,2^. A proper coordination between the repair machinery, damage tolerance, and checkpoint pathways is required for preserving DNA integrity and proper cell function^3^.

A list of 276 DDR genes was recently identified by The Cancer Genome Atlas (TCGA) DNA Damage Repair Analysis Working Group, which systematically analysed potential causes of loss of DDR function across 33 different cancer types and their consequences in human cancer^2^. This set of genes provides the basis for the mechanistic and therapeutic analysis of the role of DDR in cancer. In addition, pan-cancer studies provide evidence for factors affecting predisposition to different cancer types, highlighting rare germline cancer susceptibility variants that affect tumour suppressor genes including *ATM*, *BRCA1*, *BRCA2*, *BRIP1*, and *PALB2*^4^. These and other studies looked at somatic and germline variants and their clinical associations to help understand individual susceptibility to tumour development^5,6^. However, a large structural analysis of candidate DDR genes accumulating pathogenic germline and recurrent somatic variants is not yet available.

Concerning prostate cancer (PCa), it has been reported that genetic variations in some DDR genes not only increase the risk of the disease, but are also associated with poor prognosis and clinical outcomes at different PCa stages^7^. Previous studies found that 22.7% (34/150) of metastatic castration-resistant PCa (mCRPC) patients harbour biallelic somatic/germline variants in DDR genes, and specifically 8% had germline variants^8^. Other studies found that 11.8% (82/692) of patients with mCRPC had at least one germline variant in a gene involved in DNA repair^9^. These and other mCRPC-based studies^8–14^ highlighted *BRCA2* as the most frequently involved, and also found that a high proportion of those patients presented a second “hit” somatic aberration in either of two forms: loss-of-function mutation or a gene-copy-loss^9,15,16^. The same authors suggested that only half of the patients could be detected by germline variants in DDR genes. Based on these studies, there is an evident need to test or examine routinely all patients with mCRPC for the presence of germline/somatic variants in DDR genes.

In this article, we study the different distributions of the following types of variants: germline benign, pathogenic, variants of unknown significance (VUS), and somatic recurrent variants. We analyse them in three different contexts. The first is Pfam domains, defined as commonly-recurring regions of protein sequence, as identified in the Pfam database^17^. These domains tend to have a functional role, and hence any disruption may affect the protein’s biological function. The second context is within the three-dimensional (3D) structures of the proteins, where available, as obtained from the Protein Data Bank (PDB)^18^. Finally, we look at the variants in protein interaction interfaces.

We find that the percentage of somatic variants accumulated in functional domains and protein interactions interfaces, is higher than for germline pathogenic variants and VUS, providing a synergistic effect to damage the protein function. In addition, for the ATM and BRCA2 proteins, we identified 3D clusters and hotspot regions that accumulate pathogenic germline and recurrent somatic variants from primary and metastatic tumours, showing the synergistic effect of a few mutations located in specific regions and contributing to oncogenesis. With this analysis, we provide a better characterisation of the most recurrent affected functional regions in DDRs, which can help the understanding of an individual’s susceptibility to tumour development.

## Results

### DDR genes accumulate more germline variants than their non-DDR interactors in the NetDDR network

The DDR protein–protein interaction annotations were retrieved from the BioGRID^19^, InWeb_IM^20^ and OmniPath^21^ databases using the Metascape tool^22^. This search resulted in a DDR interaction network (NetDDR) with 229 DDR-hits and 1,182 non-DDR-hits, joined by 38,494 edges, which corresponded to the more connected network according to Metascape^22^ (for a detailed description of NetDDR see “Materials and methods”).

**Figure 1:**
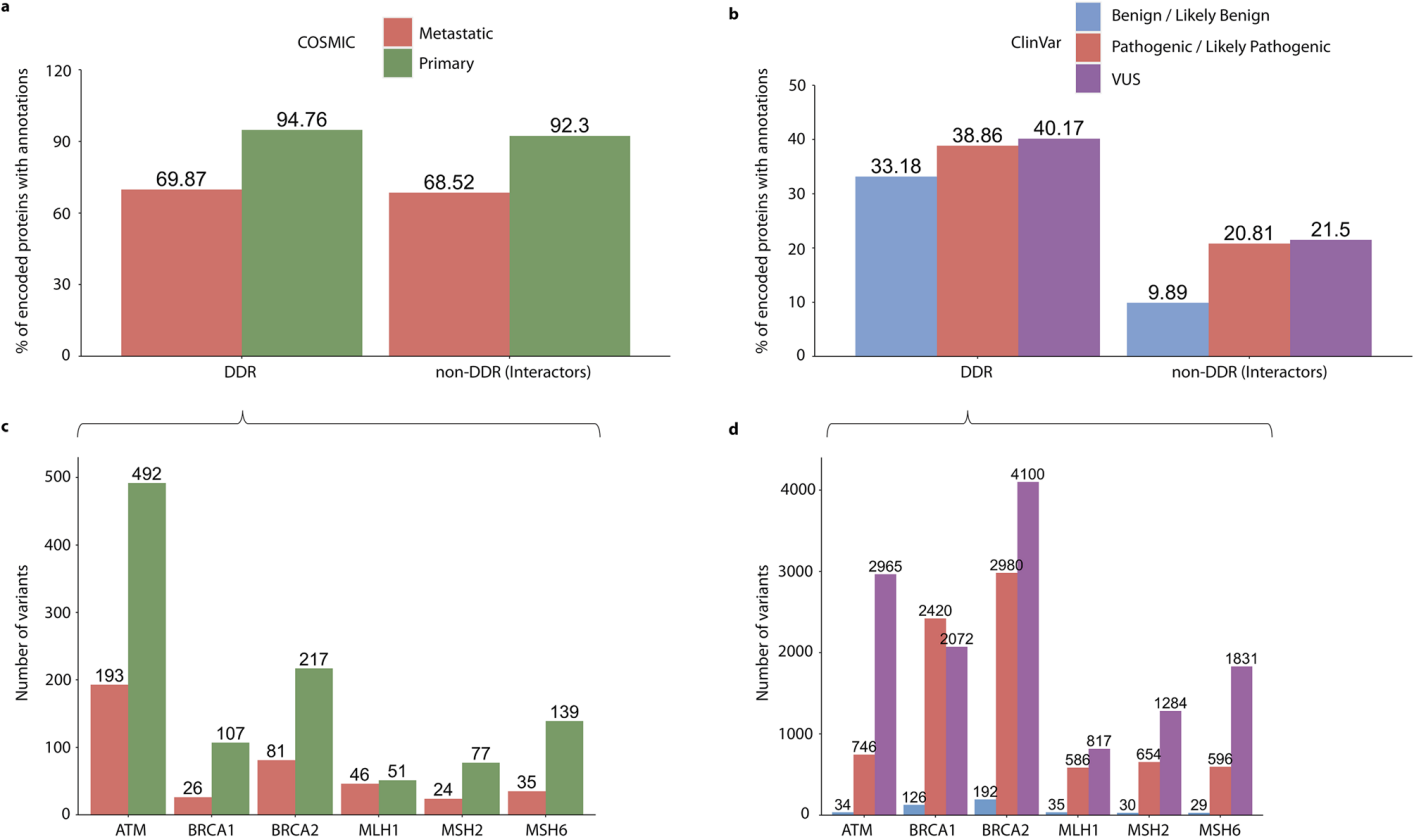
Accumulation of germline and recurrent somatic variants in DDR and non-DDR interactors in the NetDDR network. Percentages of protein-coding genes with variant annotations in COSMIC and ClinVar are shown in panels (**a**,**b**), respectively. Patterns of recurrent somatic (≥ 2 samples) and germline variants in the highly mutated DDR genes are illustrated in panels (**c**,**d**), respectively. An additional analysis of the variant distributions, normalized by protein length, is shown in Supplementary Fig. S6.

We first used the NetDDR interaction network to investigate the importance of the DDR protein-coding genes in terms of their protein interactions and accumulation of genetic variants. We found that recurrent somatic variants (i.e. occurring in more than one sample) annotated in COSMIC are found in more than 93% of both DDR and non-DDR interacting proteins in the NetDDR network (Fig. 1, panel a). However, germline variants annotated in ClinVar were identified in DDR (40%) more frequently than in non-DDR (21%) genes (Fig. 1, panel b). This finding suggests there is some bias towards the most studied genes, human variations, and phenotypes with supporting evidence annotated in the ClinVar database, whose primary source of information are published studies. In contrast, the COSMIC database documents somatic mutations in human cancers not only from published studies, but also from whole genome and exome sequencing experiments, that identify variants more homogeneously across all human genes.

In addition, the largest set of ClinVar annotations are VUS variants, followed by pathogenic and benign, thus showing another example of bias in the dataset. Other authors have observed some of the biases associated with ClinVar, in particular, the inflated pathogenic variants profiles that make it difficult to study variant penetrance and disease prevalence in endocrine tumour syndromes^23^. In the COSMIC database we found fewer variants identified in metastatic tumours than in a primary state (Fig. 1, panel a), probably due to the low number of individuals studied with advanced cancer.

Also shown in Fig. 1 are the numbers of somatic and germline variants in the cancer-predisposition genes *ATM*, *BRCA1*, *BRCA2*, *MLH1*, *MSH2*, and *MSH6* (Fig. 1, panels c and d). These DDR genes are among the most affected in the COSMIC and ClinVar datasets (Supplementary Fig. S5). The majority of recurrent somatic variants, observed in more than two samples, were from primary tumours. In addition, most germline variants in these cancer-predisposition and DDR genes correspond to pathogenic and VUS, although their ratios varied depending on the gene analysed. Besides, DDR cancer-predisposition genes are highly connected and central in the NetDDR interaction network. Indeed, the average node degree (AvrNodDeg) for these six genes is 166, while in the complete DDR-hits AvrNodDeg = 87. As expected, closeness centrality (ClossCentl), which indicates how close a node is to all other nodes in the network, is higher on average for the DDR-hits (ClossCentl = 0.46) compared to the complete NetDDR interaction network (ClossCentl = 0.43) (for more details see Supplementary Table S1). It has been suggested that mutations in regulatory regions of highly connected protein-coding genes in protein–protein interaction and regulatory networks, have a higher functional impact than those targeting peripheral genes in the network^24^.

In the sections below we also investigated the effects of protein length, domain composition, and protein 3D structure on the accumulation of germline and somatic variants in DDR protein-coding genes.

### DDR genes show a different pattern of accumulation of germline and somatic variants as a function of protein length

**Figure 2:**
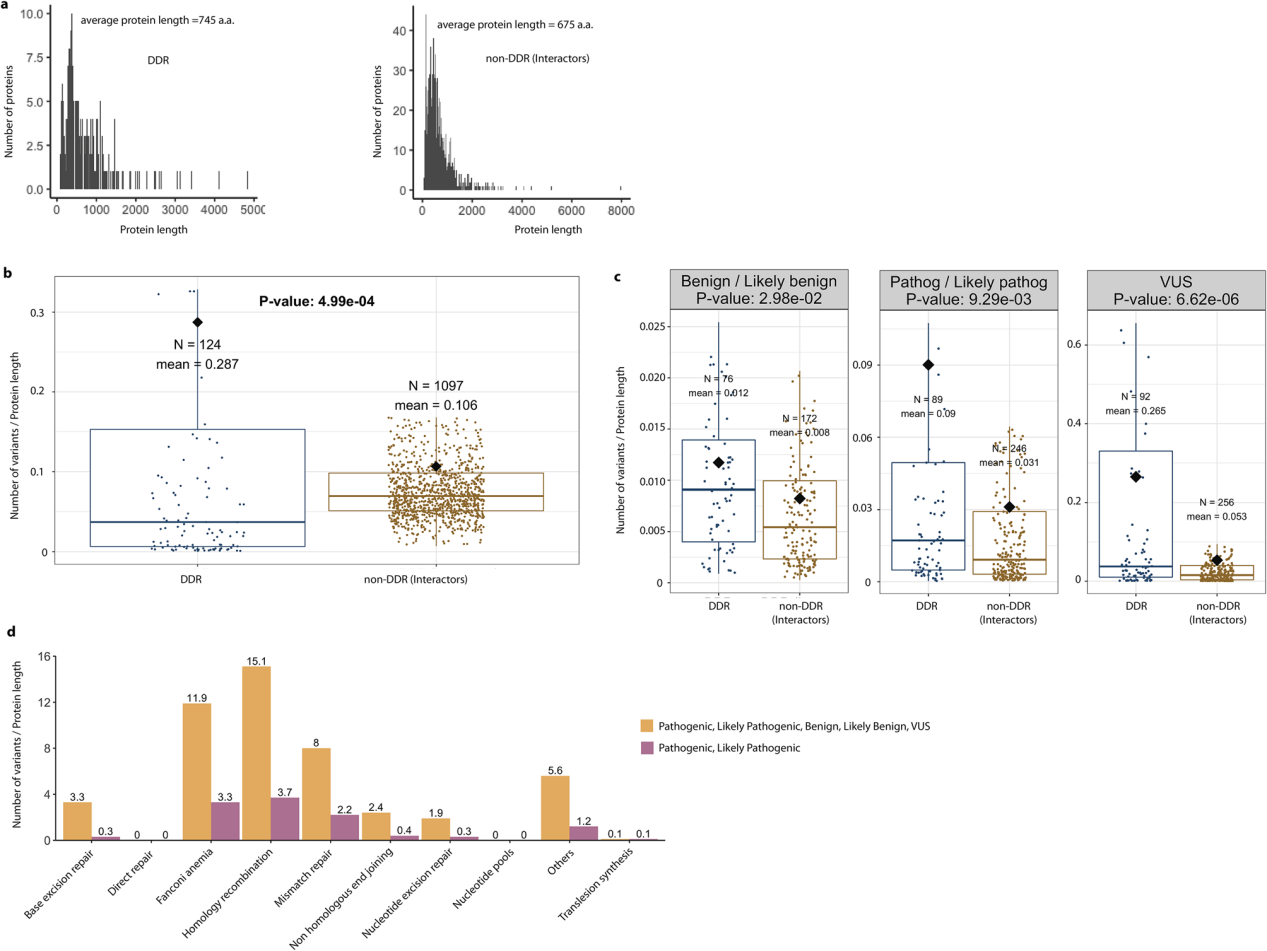
Distributions of germline variants in DDR and non-DDR interactors. Panel (**a**) shows the distribution of protein lengths for DDR and non-DDR proteins. Their average lengths are similar, being 745 a.a and 675 a.a, respectively. Boxplots show the contrasting patterns of germline variants as a function of protein length in the DDR and non-DDR proteins (panel **b**), and as categorized into benign, pathogenic, and VUS (panel **c**). Outliers in panel b and c were removed here for clarity, but included in the T-test statistical analysis. Some genes occur in two or three of the categories in panel (**c**), hence the sum of the N values shown is higher than the N in the corresponding plot in panel b which is the number of unique DDR genes. All boxplots depict the first and third quartiles as the lower and upper bounds of the box, with a thicker band inside the box showing the median value, and whiskers representing 1.5 × the interquartile range. Panel d shows the accumulation of germline variants in biological pathways related to the 229 DDR genes.

First, we studied the distribution of the protein length in the DDR and non-DDR groups (Fig. 2, panel a). Although the average protein lengths for DDR (745 a.a.) and non-DDR (675 a.a.) are very similar, we compared the accumulation of somatic and germline variants in the most affected cancer-predisposition genes *ATM*, *BRCA1*, *BRCA2*, *MLH1*, *MSH2*, and *MSH6* as a function of protein length (Supplementary Fig. S6). These cancer-predisposition genes show a similar pattern of accumulation of somatic and germline variants with and without normalization by protein length.

**Figure 3:**
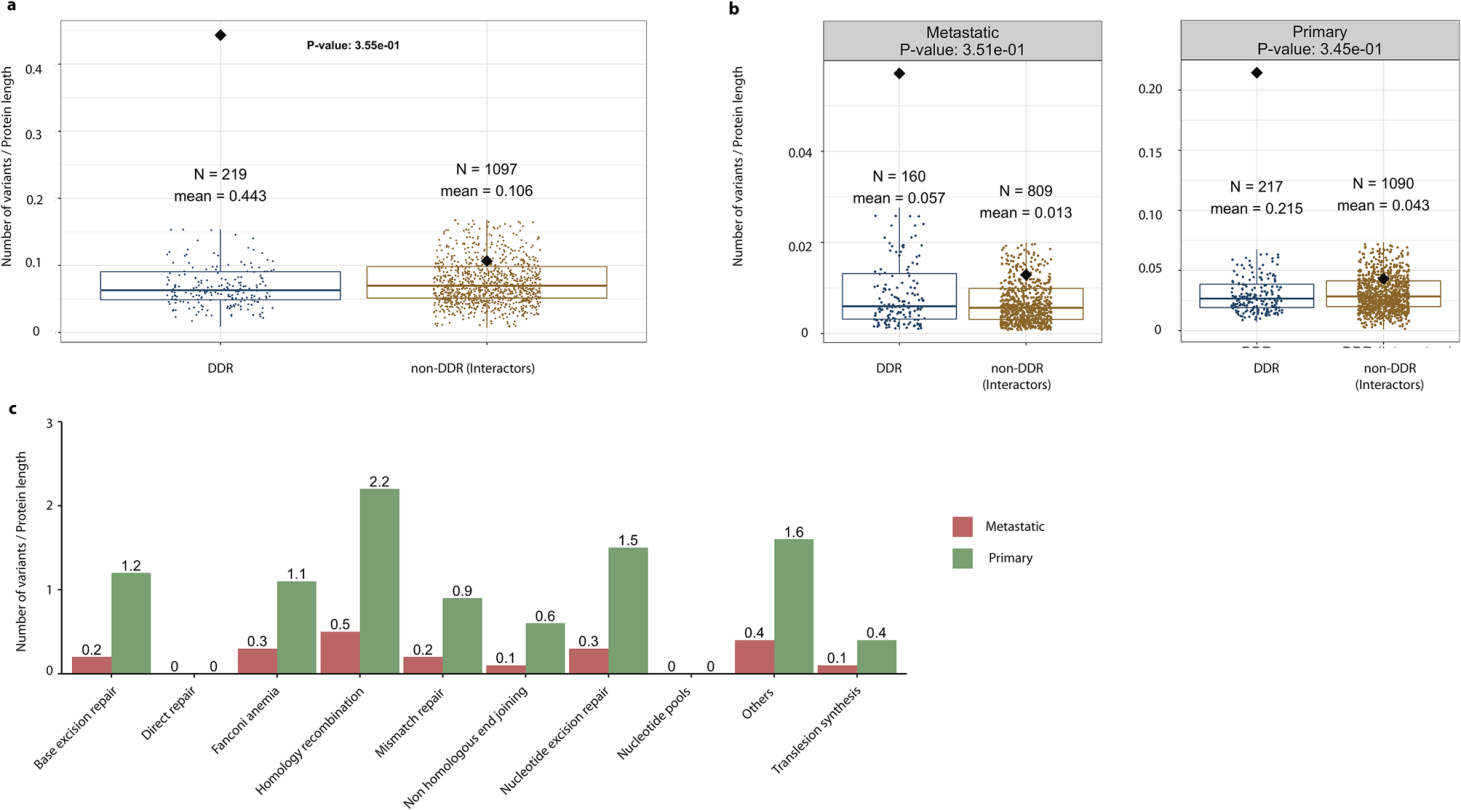
Distributions of somatic variants in DDR and non-DDR interactors. Boxplots showing the contrasting patterns in somatic variants as a function of protein length in DDR and non-DDR (panel **a**), and as categorized into metastasis and primary tumours (panel **b**). Outliers in panels (**a**,**b**) were removed for clarity, but retained for the T-test statistical analysis. Some genes occur in one or two of the categories in panel **b**, hence the sum of the N values is higher than in the corresponding plot in panel (**a**). The N in panel (**a**) indicates the number of unique DDR genes. All box plots depict the first and third quartiles as the lower and upper bounds of the box, with a thicker band inside the box showing the median value and whiskers representing 1.5 × the interquartile range. Panel (**c**) shows the accumulation of somatic variants in different biological pathways associated with the 229 DDR genes. For better visualization of the barplot we excluded *TP53,* which has a large number of recorded variants.

We also analysed the distribution of germline and somatic variants, normalized by protein length, using the complete dataset of DDR and non-DDR proteins (Fig. 2 and Fig. 3, respectively). The proteins in our dataset range in length from 44 a.a. (TMSB4X) to 7,968 a.a. (OBSCN). The boxplot in Fig. 2, panel b, shows that the mean number of germline variants per protein length in the DDR proteins, according to ClinVar database, is 0.287, which is significantly higher than the mean of 0.106 for the non-DDR (P-value = 5.0 × 10^–4^) proteins.

Panel c shows the differences when the variants are grouped by the different categories (i.e., benign/likely benign, pathogenic/likely pathogenic, and VUS). A few DDR proteins appear above the 75th percentile, indicating accumulation of a high number of germline variants per protein length (i.e. BRCA1, BRCA2, MLH1, MSH2, MSH6) (Supplementary Fig. S5). So, we hypothesize that the accumulation of a large number of ClinVar annotations in these DDRs could bias research findings or limit the generalizability of the results. Moreover, Fig. 2, panel c, shows a greater variability in the DDR distributions and also shows that the medians (at 95% confidence interval) tend to be larger than for the non-DDR group, with P-values being 3.0 × 10^–2^ for benign, 9.3 × 10^–3^ for pathogenic, and 6.6 × 10^–6^ for VUS germline variants.

In addition, we analysed the accumulation of germline and somatic variants in the different pathways where DDRs are involved. We found that the Homology Recombination, Fanconi Anaemia, and Mismatch Repair pathways are the most affected by germline mutations (Fig. 2, panel d). The analysis of recurrent somatic variants annotated in COSMIC, also indicated the same affected pathways (Fig. 3, panel c).

The boxplots in Fig. 3 (panels a and b) show similar medians and dispersion of the somatic variants, according to the COSMIC database, per protein length in DDR and non-DDR groups (P-value = 0.36; metastatic: P-value = 0.35, primary: P-value = 0.35). However, this pattern of variability is different to the one previously observed in the germline variants annotated in ClinVar. In this case, *ATM*, *ATRX*, *CHEK2*, *ERCC2*, *MLH1*, *MSH6*, and *SMARCA4*, among other genes, are representative DDRs above the 75th percentile that indicate accumulation of somatic variants from the COSMIC database. Moreover, according to panel c in Fig. 3, only the Nucleotide Excision Repair (NER) pathway appears to be more affected by somatic variants in primary tumours than by germline pathogenic variants (Fig. 2, panel d). Therefore, our analyses of accumulation of somatic variants in the NER pathway coincide with the results by other authors in that this pathway shows an increased contribution of a somatic mutational pattern (COSMIC mutational signature 8) recurrently observed in various cancer types^25^. So, the disruption of this pathway could potentially drive carcinogenesis and accelerate aging.

### DDR germline and somatic variants occur differently within Pfam domains and protein interaction interfaces

**Figure 4:**
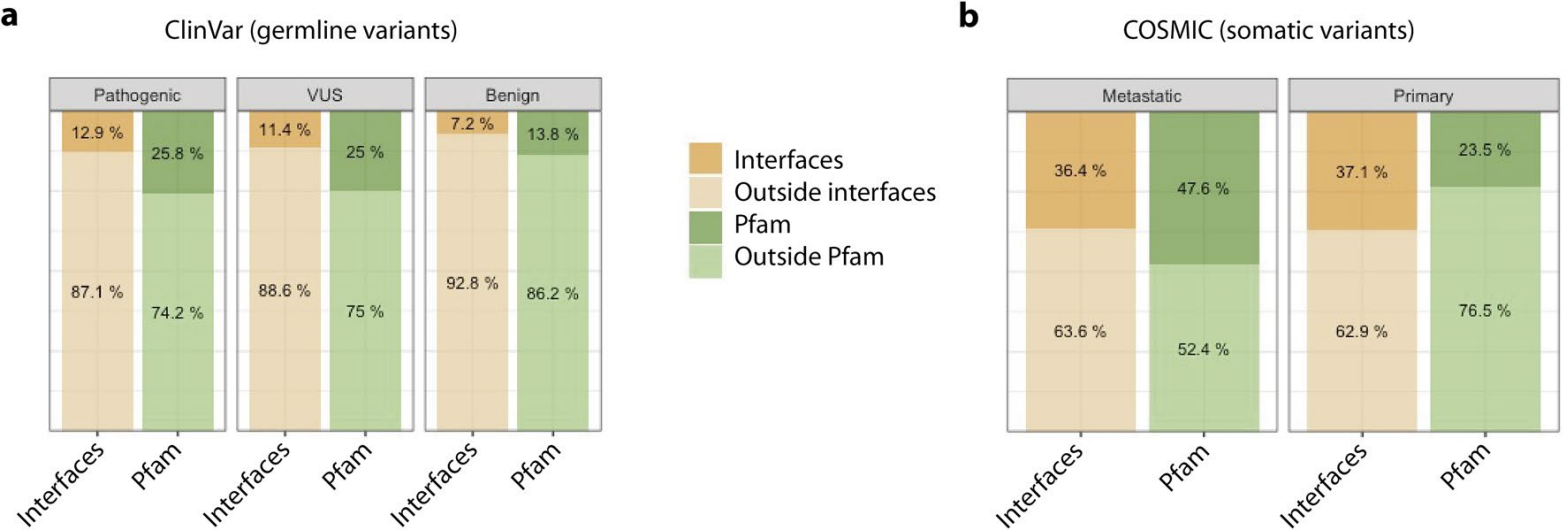
Accumulation of germline and somatic variants in Pfam domains and protein interaction interfaces. Panel (**a**) shows the percentage of pathogenic, VUS and benign germline variants extracted from the ClinVar database across interfaces and Pfam domains. Panel (**b**) shows the percentage of metastatic and primary somatic variants extracted from COSMIC across interfaces and Pfam domains.

We also investigated the occurrence of DDR germline and somatic variants according to the ClinVar and COSMIC databases, respectively, in Pfam domains and protein interaction interfaces. The curated list of germline and recurrent (≥ 2 samples) somatic variants were annotated using the Structure-PPi system^26^ and the results are summarized in Fig. 4 and in Supplementary File 2 and Supplementary Table S2. The number of variants annotated in the different classes were: for germline variants (1) 10,301 pathogenic and likely pathogenic, (2) 1,117 benign and likely benign, and (3) 28,248 VUS, whereas for somatic variants (1) 5,795 variants in primary tumours and (2) 2,030 variants in metastasis. These results indicate that germline variants in the pathogenic and VUS classes have a similar distribution across Pfam domains (P-value = 0.13) (Fig. 4, panel a). In particular, they possess a very similar percentage of variants mapped onto protein interaction interfaces (12.9% and 11.4%, respectively), and higher than in the case of benign variants (7.2%). Interestingly, the percentage of somatic variants in metastatic tumours found in both Pfam domains and protein interaction interfaces (Fig. 4, panel b), is significantly higher than in germline pathogenic (P-value < 0.0001) and VUS variants (P-value < 0.0001) (Fig. 4, panel a). The 47.6% metastatic variants affecting Pfam domains are double the value of 25% for germline pathogenic and VUS variants (Fig. 4, panel a and b). Otherwise, the percentage of metastatic variants affecting protein interaction interfaces (36.4%) is threefold higher than the value of 13% to 11% in pathogenic and VUS germline variants, respectively.

The most affected Pfam domains by germline pathogenic variants were: MutS_III (Pfam code: PF05192, 371 variants), MutS domain V (Pfam code: PF00488, 323 variants), and BRCA2 (Pfam code: PF00634, 224 variants). MutS domains are found in human MSH2/MSH6 proteins implicated in non-polyposis colorectal carcinoma (HNPCC), while *BRCA2* is a known tumour suppressor gene. For the BRCA2 or FANCD1 proteins, their association with the Fanconi anaemia (FANC) protein complex is well known^27^. Other Pfam domains affected with germline pathogenic variants are: BRCA2-helical (Pfam code: PF09169, 159 variants), BRCA2-OB1 (Pfam code: PF09103, 116 variants), PTEN-C2 (Pfam code: PF10409, 110 variants), BRCA1 C-terminus (Pfam code: PF00533, 108 variants), P53 (Pfam code: PF00870, 107 variants) and MutS domain IV (Pfam code: PF05190, 105 variants). It is very interesting that the DDR pathogenic germline variants in functional domains tend to occur in the known tumour suppressors genes *P53*, *BRCA2* and *PTEN*. But as we commented before, this observation would suggest some bias towards the most studied genes and phenotypes with supporting evidence annotated in the ClinVar database.

On the other hand, Pfam domains affected by somatic variants identified in metastatic tumours also included the known tumour suppressor genes *P53* and *PTEN*: P53 DNA-binding (Pfam code: PF00870, 408 variants), PTEN-C2 (Pfam code: PF10409, 44 variants) and P53 tetramer (Pfam code: PF07710, 27 variants). A description of the affected Pfam domains discussed in this article and the associated functions is presented in Table 1.

**Table 1:** List of Pfam domains affected by germline and somatic mutations in the DDR family.

Pfam ID	Description	Cellular functions of domain
PF05192	MutS_III	DNA mismatch repair and recombination
PF05190	MutS domain IV	Involved in mismatch repair
PF00488	MutS domain V	Involved in mismatch repair and DNA binding
PF00634	BRCA2 repeat	Involved in the repair of chromosomal damage with an important role in the error-free repair of DNA double strand breaks. Breast cancer susceptibility protein
PF09169	BRCA-2_helical	Binds DSS1 protein
PF09103	BRCA-2_OB1 (BRCA2 oligonucleotide/oligosaccharide-binding domain 1)	Binds DSS1 protein
PF09121	Tower domain	Essential for appropriate binding of BRCA2 to DNA. Has an important role in the tumour suppressor function of BRCA2
PF09104	BRCA-2_OB3 (BRCA2, oligonucleotide/oligosaccharide-binding domain 3)	Binds DSS1 protein
PF10409	PTEN-C2	Plays a central role in membrane binding, productively positions the catalytic part of the protein onto the membrane
PF00870	P53	DNA-binding domain
PF07710	P53 tetramer	
PF00097	zf-C3HC4 (Zinc finger RING type)	Zinc fingers bind DNA, RNA, protein and/or lipid substrates. RING fingers play a key role in the ubiquitination pathway
PF12820	BRCT_assoc (serine-rich associated with BRCT)	No specified function
PF00533	BRCT (BRCA1 C-terminus)	Involved in cell cycle checkpoint functions responsive to DNA damage. A marker of breast cancer susceptibility
PF11640	TAN (Telomere-length maintenance and DNA damage repair)	Regulates responses to DNA double-strand breaks (EC:2.7.11.1)
PF02259	FAT (A novel domain in PIK-related proteins; i.e. ***F***RAP, ***A***TM, and ***T***RRAP)	Still need to be elucidated experimentally. Usually involved in protein–protein interactions
PF00454	PI3_PI4_kinase (Phosphatidylinositol 3- and 4-kinase)	Involved in cell growth, proliferation, differentiation, motility, survival and intracellular trafficking, which in turn are involved in cancer
PF02260	FATC (A novel motif at the extreme C-terminus of PIK-related proteins)	Still need to be elucidated experimentally. Usually involved in protein–protein interactions
PF00730	HhH-GPD (HHH-GPD superfamily base excision DNA repair protein)	Involved in DNA repair functions (i.e. endonuclease III, DNA glycosylase, and methyl-CPG binding protein)
PF00633	HHH (Helix-hairpin-helix motif)	DNA-binding domain
PF14815	NUDIX_4 (NUDIX domain)	A/G‐specific adenine glycosylases. Involved in DNA base‐pair mismatch repair

Overall, these observations agree with the hypothesis of two-hit events: first a germline variant produces a predisposition to develop a tumour, while a second somatic event increases this probability in a specific organ and triggers tumour development.

### Co-localization of pathogenic germline and somatic variants in protein sequence and 3D structure in DDR genes ATM, BRCA1, BRCA2, and MUTYH

In a previous work, we described *ATM*, *BRCA1, BRCA2*, and *MUTYH* as recurring genes with mutations in the PROREPAIR-B PCa cohort^14^. Here, as use cases in the DDR research, we studied these genes and analysed more pathogenic germline variants identified in different cohorts of advanced PCa reported in the literature (Table 2), recurrent somatic and germline variants in PCa, as well as hotspot positions in different tumour types collected from cBioPortal (https://www.cbioportal.org) (Supplementary File 1), and also, the TCGA-PanCancer study of pathogenic germline variants in 10,389 adult cancers^28^.

**Table 2:** Pathogenic germline variants identified in mCRPC cohorts and affecting the PCa relevant genes *ATM*, *BRCA1*, *BRCA2* and *MUTYH*.

Publication	ATM*	BRCA1*	BRCA2*	MUTYH*	Population (n)	%*BRCA2* mutated
Robinson et al.^8^	8	4	11	–	150	7.3
Pritchard et al.^9^	11	6 (3)	37 (24)	–	692	5.35
Na et al.^10^	6	2	11	–	313	3.51
Mijuskovic et al.^11^	3	0	4	–	139	2.88
Annala et al.^12^	1	1	16 (13)	–	202	7.92
Antonarakis et al.^13^	3	1	5 (3)	–	172	2.91
PROREPAIR-B^14^	8 (5)	4	14 (13)	13 (3)	419	3.34
cBioPortal (recurrent)	10	2	16	1	1,556 ^(a)^	8.0
cBioPortal (hotspots)	3	3	6	3	44,284 ^(b)^	4.0

(*) Numbers indicate the total variants identified in each study and in parenthesis the non-identical germline variants. (a) From 11 PCa studies in the non-redundant dataset at cBioPortal. (b) From 176 different studies in the non-redundant dataset at cBioPortal. Details about the PCa populations in cBioPortal are provided in Supplementary File 1.

**Figure 5:**
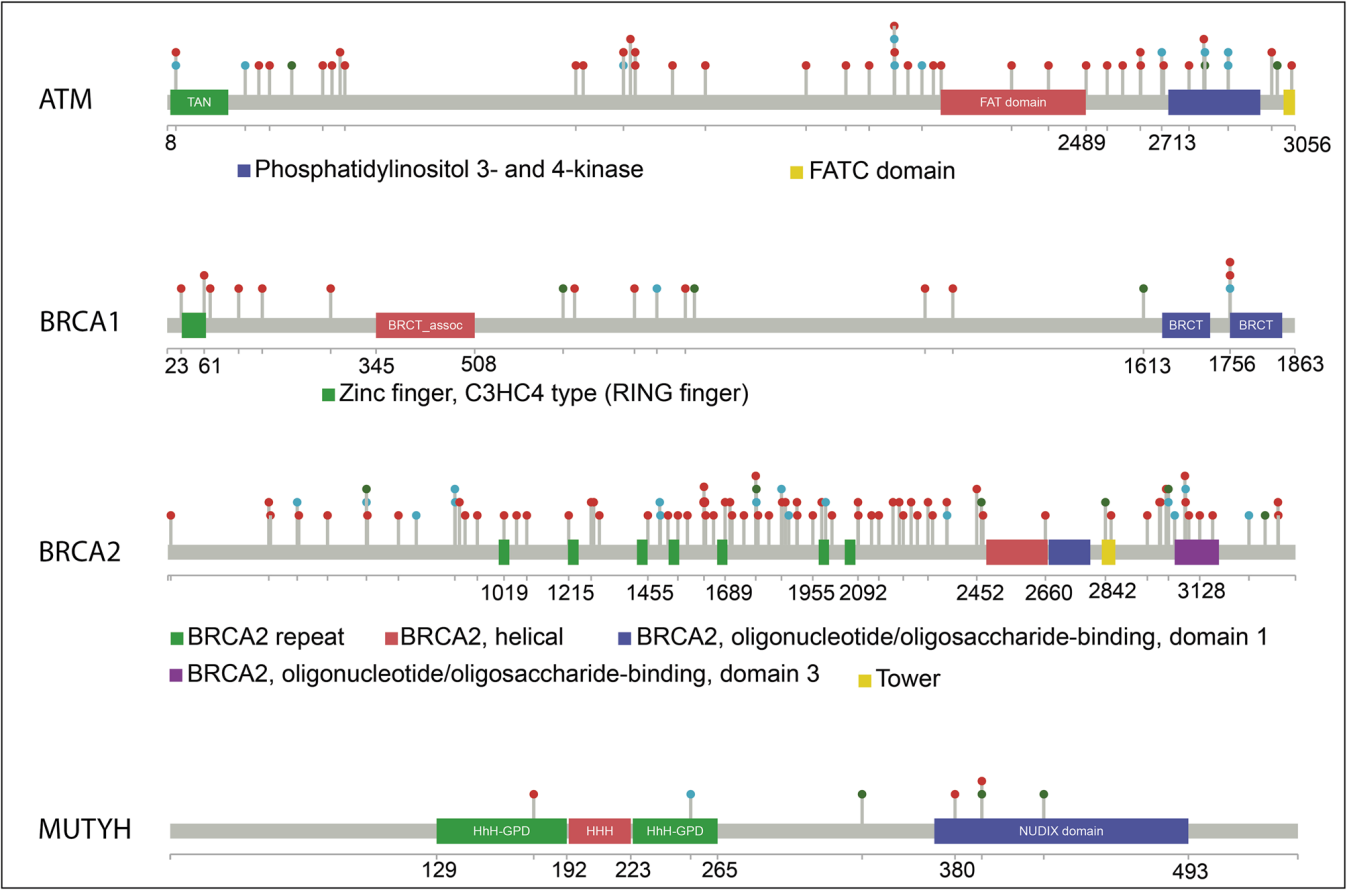
Lollipop diagrams of germline and somatic variants in ATM, BRCA1, BRCA2 and MUTYH. The locations of different types of variants along the protein sequences are indicated by the coloured “lollipops”. The variants are coloured: red for pathogenic germline variants identified in different cohorts of advanced PCa, cyan for recurrent germline and somatic variants in PCa, and green for hotspot positions in different tumour types.

Figure 5 shows the distribution of these different types of variants across each protein sequence. We observe that the vast majority of variants in ATM, BRCA1, and BRCA2 are localized in flexible and/or intrinsically disordered regions (IDRs), outside Pfam domainss (Fig. 5, panel a). The IDRs are common in protein interaction interfaces. In fact, the NetDDR interaction network revealed a high number of interactions involving these specific DDRs: ATM (AvrNodDeg = 228), BRCA1 (AvrNodDeg = 299), BRCA2 (AvrNodDeg = 94), and MUTYH (AvrNodDeg = 17). Interestingly, the high number of interactions in ATM and BRCA1, in comparison with the moderate number in BRCA2 and MUTYH, coincide with a higher predicted consensus disorder content in ATM and BRCA1 (16.4% and 82.3%, respectively) than in BRCA2 and MUTYH (3.2% and 7.5%, respectively). The percentages of disordered regions are as given in the MobiDB database (https://mobidb.bio.unipd.it).

**Figure 6:**
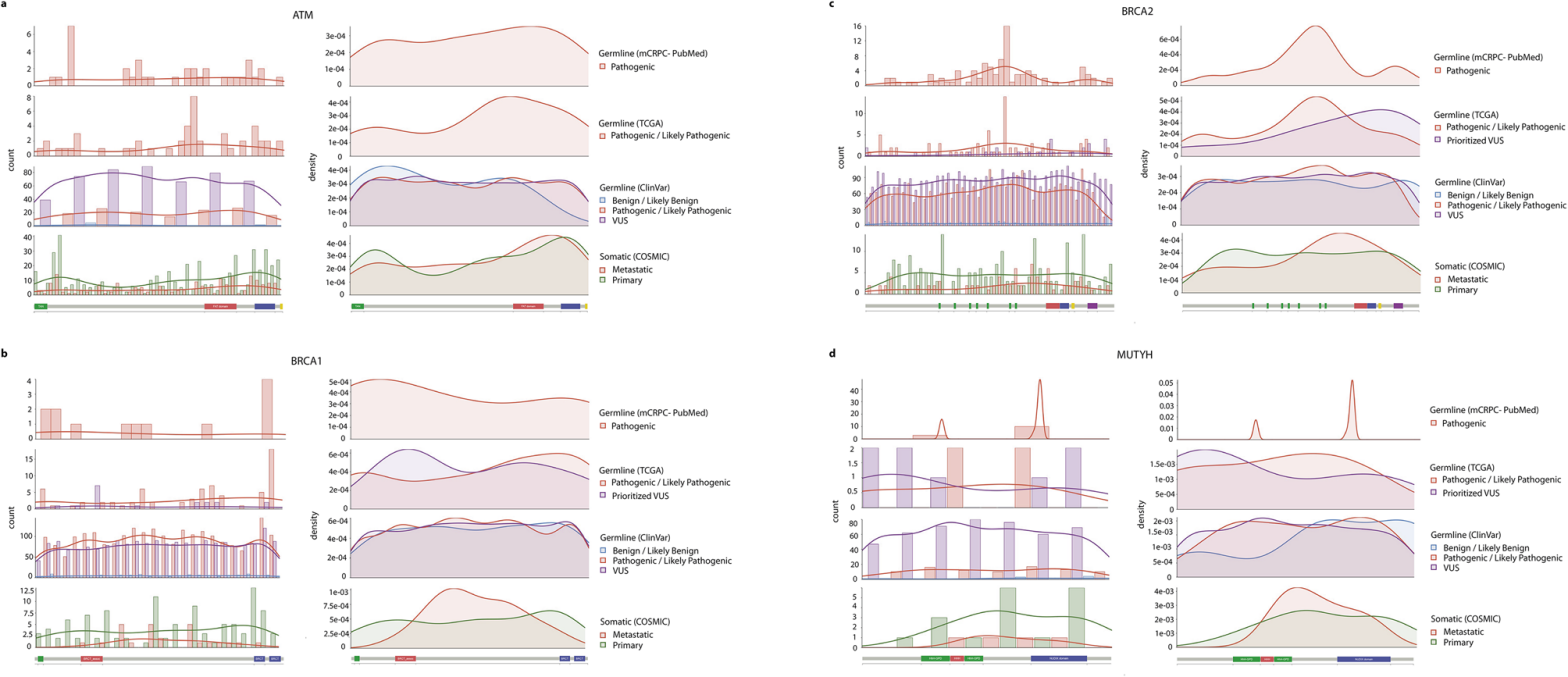
Co-localization of germline and somatic variants in ATM, BRCA1, BRCA2 and MUTYH. Panel (**a**): Histograms and density distributions for different types of variants in ATM. The histograms in the left-hand column show the counts of each variant type in bins of 75 residues along the sequence. They show: germline variants identified in different cohorts of mCRPC collected from the literature (PubMed), germline variants from TCGA, germline variants from ClinVar, and somatic variants from COSMIC. Where more than one type of variant is plotted the widths of the bars in each bin are reduced accordingly. To the right of each histogram are the equivalent density distributions, as computed by smoothing the histogram bars. A small version of the Pfam domain layout is also shown beneath the histograms and density distributions. Panels (**b**–**d**) show the data for BRCA1, BRCA2, and MUTYH, respectively.

Moreover, we observed a few cases where PCa germline and recurrent somatic variants either co-localize at the same amino acid position, or are close neighbours (e.g., red and cyan dots in Fig. 5). Based on this finding, we expanded the analysis and compared the distribution of germline and somatic variants from different datasets as shown in Fig. 6 (i.e., germline variants identified in different cohorts of mCRPC collected from the literature (PubMed), germline variants from TCGA, germline variants from ClinVar, and somatic variants from COSMIC). Germline and somatic variants are distributed along the full length of the protein sequence, although the shape of histograms and density plots is not symmetrical and shows different peaks, which indicate accumulation of variants in defined regions of the protein. Table 3 shows significant differences (P-value < 0.05) between the pathogenic germline variants versus their benign and VUS counterparts in BRCA2. The same significant tendency was observed in BRCA2 for the distribution of VUS versus benign, both in the ClinVar dataset. No significant differences were observed in the distribution of somatic variants between primary and metastatic tumours.

**Table 3:** Statistical analysis of the distribution of the variants across the protein sequence.

		**ATM**	**BRCA1**	**BRCA2**	**MUTYH**
COSMIC Metastatic/Primary	D	0.078415	0.25953	0.15202	0.25
	P-value	0.3615	0.1195	0.1309	0.9853
ClinVar Pathogenic/VUS	D	0.029031	0.033094	0.052161	0.089771
	P-value	0.6966	0.1733	**0.000167**	0.5426
ClinVar Pathogenic/Benign	D	0.19303	0.076781	0.15275	0.36809
	P-value	0.1771	0.4802	**0.0004421**	0.1726
ClinVar VUS/Benign	D	0.19993	0.054376	0.11721	0.39808
	P-value	0.1361	0.8739	**0.01295**	0.08924

Statistical significance of the differences between the density distributions in Fig. 6, as calculated using the GLDEX package (https://CRAN.R-project.org/package=GLDEX). Statistically significant differences (shown in bold) are indicated where the P-value is less than 0.05.

In ATM (Fig. 6, panel a), the pathogenic germline variants from different mCRPC cohorts, TCGA, and ClinVar datasets show a tendency to accumulate in the interaction region with the NF-κ B essential modulator IKBKG (NEMO) (a.a. 1960–2565; IntAct accessions: EBI-495465 and EBI-81279), that overlaps a flexible region flanking the FATC domain (Pfam code: PF02260). The density of benign variants in this interaction region is different from pathogenic and VUS. Somatic variants from COSMIC in primary and metastatic tumours also tend to accumulate in the same interaction region. A different scenario is observed in BRCA1 (Fig. 6, panel b) and BRCA2 (Fig. 6, panel c). In BRCA1, the pathogenic germline variants from ClinVar show a uniform distribution, but in different cohorts of PCa they accumulate over a flexible region connecting a RING type Zinc finger (zf-C3HC4; Pfam code: PF00097) and the serine-rich region associated with BRCT (BRCT_assoc; Pfam code: PF12820) N-terminal domains. By contrast, pathogenic variants from TCGA accumulate in the C-terminus, which is the interaction region with FANCJ (IntAct accessions: EBI-3509650 and EBI-349905). In BRCA2, pathogenic variants in different PCa cohorts, TCGA, and ClinVar datasets show a peak over the central flexible region including BRCA2 repeats (Pfam code: PF00634), that constitute the interaction region with RAD51 (IntAct accessions: EBI-79792 and EBI-15557721). Somatic variants in metastatic tumours accumulate in the linker and flexible region between central BRCA2 repeats and the C-terminus of the protein. These findings reinforce the idea of a synergistic effect between germline and somatic variants, and that somatic events tend to accumulate in protein interaction regions such as IDRs. Also, according to the mutational data available, for some DDR members it is possible to identify protein regions that accumulate pathogenic variants versus benign and VUS. Remarkably, disrupting or impairing these protein interactions is likely to have a marked impact on their function, since these cancer-predisposition genes are highly connected and central in the NetDDR interaction network.

**Figure 7:**
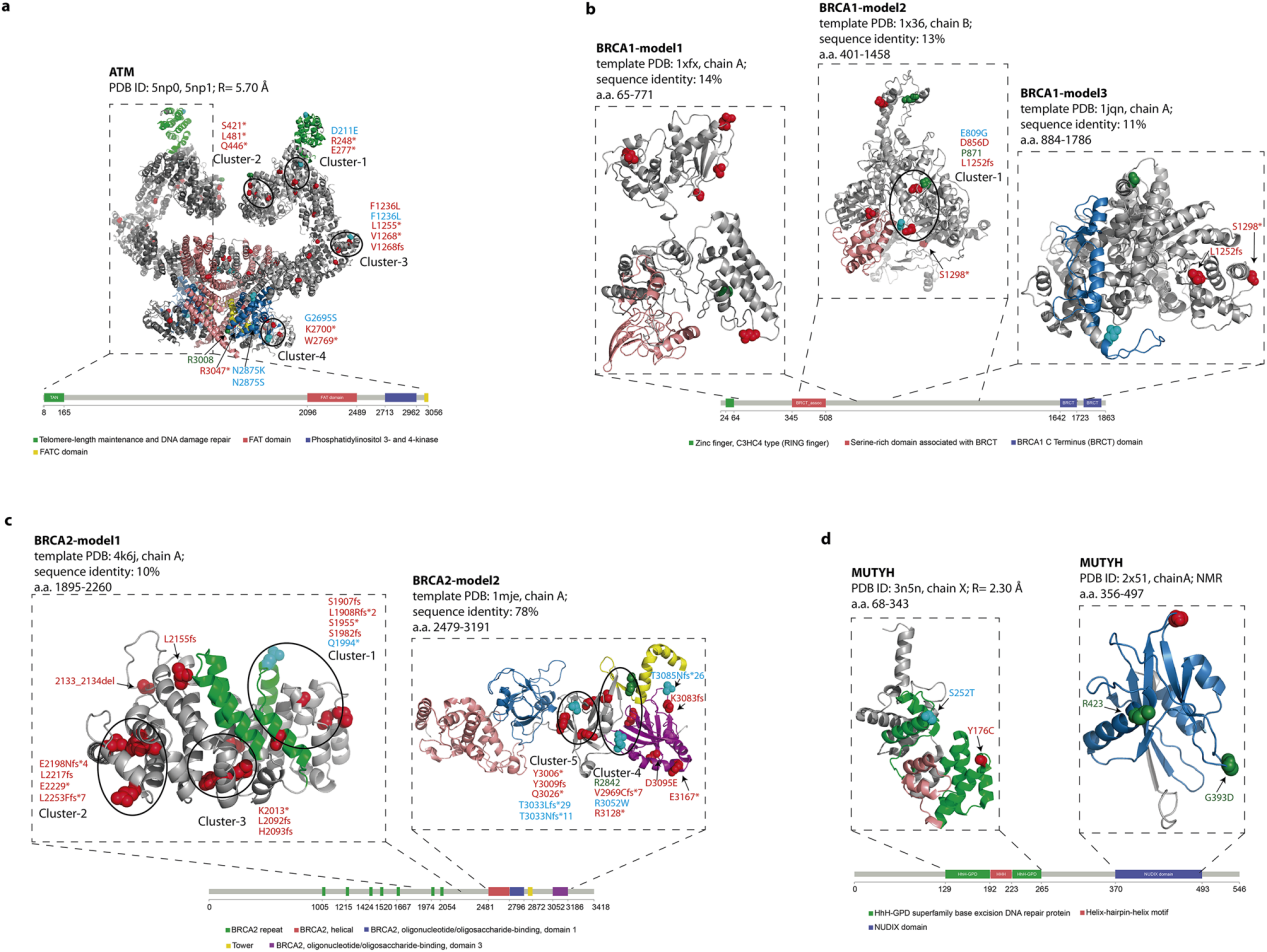
Mapping of germline and somatic variants onto protein 3D structures. The spatial 3D clusters in ATM (panel **a**), BRCA1 (panel **b**), BRCA2 (panel **c**), and MUTYH (panel **d**) are highlighted. Pathogenic germline variants are represented in red, recurrent somatic and germline in PCa in cyan, and hotspot positions in different tumour types in green. The spatial 3D clusters were calculated using the Mutation3D program (http://mutation3d.org)^29^.

We also studied the co-localization of variants that are discontinuous along the sequence but proximal in the protein 3D structure. The pathogenic germline variants in mCRPC, recurrent germline and somatic variants, and hotspot positions in different tumour types were mapped onto ATM, BRCA1, BRCA2, and MUTYH available structures (Fig. 7), in order to find 3D-clusters of variants. We identified different clusters containing at least three individual positions within spheres with a 15-30 Å diameter that accommodate germline and somatic variants (Fig. 7 and Table 4). Variants included in the same 3D-cluster can be considered part of a continuum of cancer-promoting variants, each with a relatively small but additive effect.

**Table 4:** List of 3D clusters identified in the protein structure of ATM, BRCA1, BRCA2 and MUTYH.

Gene	Cluster-ID	Variant	Description
ATM	cluster 1 (diameter = 16.1 Å; P-value = 4 × 10–4)	p.D211E	PCa recurrent somatic variant
		p.R248*	Pathogenic germline variants
		p.E277*	Pathogenic germline variants
	cluster 2 (diameter = 15.4 Å; P-value = 3 × 10–4)	p.S421*	Pathogenic germline variants
		p.Q446*	Pathogenic germline variants
		p.L481*	Pathogenic germline variants
	cluster 3 (diameter = 18.7 Å; P-value = 9.9 × 10–5)	p.F1236L	PCa germline variants
		p.L1255*	PCa germline variants
		p.V1268*	PCa germline variants
		p.V1268fs	PCa germline variants
		p.F1236L	Recurrent somatic variant
	cluster 4 (diameter = 12.1 Å; P-value = 1 × 10–5)	p.K2700*	PCa pathogenic germline variants
		p.W2769*	PCa pathogenic germline variants
		p.G2695S	Recurrent somatic variant
BRCA1	cluster 1 (diameter = 23.1 Å; P-value = 1 × 10–4)	p.L1252fs	Pathogenic germline variant
		p.D856D	PCa variant
		p.E809G	Recurrent somatic variant
		p.P871	Hotspot position
BRCA2	cluster 1 (diameter = 24.2 Å; P-value = 1 × 10–4)	p.S1907fs	Pathogenic germline variants
		p.L1908Rfs*2	Pathogenic germline variants
		p.S1955*	Pathogenic germline variants
		p.S1982fs	Pathogenic germline variants
		p.Q1994*	PCa recurrent somatic variant
	cluster 2 (diameter = 22.9 Å; P-value = 9.9 × 10–5)	p.E2198Nfs*4	Pathogenic germline variants
		p.L2217fs	Pathogenic germline variants
		p.L2253Ffs*7	Pathogenic germline variants
		p.E2229*	Pathogenic germline variants
	cluster 3 (diameter = 22.8 Å; P-value = 9.9 × 10–5)	p.L2092fs	Pathogenic germline variants
		p.H2093fs	Pathogenic germline variants
		p.K2013*	Pathogenic germline variants
	cluster 4 (diameter = 25.5 Å; P-value = 9.9 × 10–5)	p.V2969Cfs*7	Pathogenic germline variants
		p.R3128*	Pathogenic germline variants
		p.R3052W	PCa recurrent somatic variant
		p.R2842	Hotspot position
	cluster 5 (diameter = 16.8 Å; P-value = 9.9 × 10–5)	p.T3033Lfs*29	PCa recurrent germline
		p.T3033Nfs*11	Recurrent somatic
		p.Y3006*	Pathogenic germline variants
		p.Y3009fs	Pathogenic germline variants
		p.Y3009fs	Pathogenic germline variants
		p.Q3026*	Pathogenic germline variants

In the ATM protein (Fig. 7, panel a), according to the low-resolution Cryo-EM structures (PDB ID: 5np0, 5np1 at 5.70 Å resolution; a.a. 1–3056) (Table 5), we identified four 3D-clusters which are listed in Table 4. We found other pairs of pathogenic and recurrent variants that were close to each, but without having a significant P-value (P-value ≥ 0.05) as computed by the mutation3D method^29^. The mutation3D program computes significance by using an iterative bootstrapping algorithm to calculate a background distribution of cluster sizes arising from a random placement of an equivalent number of substitutions in the selected protein structure. For each cluster in the input data, P-values are computed empirically as the percentile rank of its “maximum cluster diameter or CL” among all for randomized clusters containing the same number of amino acid substitutions (see “Materials and methods”). As an example, the PCa pathogenic germline variant p.R3047*, recurrent somatic variants p.N2875K and p.N2875S, and the hotspot position R3008 in different tumour types, are within a sphere of diameter = 25.6 Å in the ATM protein 3D-structure (Fig. 7, panel a).

**Table 5:** List of the 3D structures of the key DDR proteins available from the PDB database.

	Region	Method	PDB id	Resolution	Chains
ATM	1–3056	Cryo-EM	5np0	5.70 Å	A/B
	1–3056	Cryo-EM	5np1	5.70 Å	A
	3024–3030	NMR	6hka	–	A
BRCA1	1–110	NMR	1jm7	–	A
	1646–1859	X-ray	1jnx	2.50 Å	X
	1646–1859	X-ray	1n5o	2.80 Å	X
	1755–1863	NMR	1oqa	–	A
	1646–1859	X-ray	1t15	1.85 Å	A
	1646–1859	X-ray	1t29	2.30 Å	A
BRCA2	1517–1551	X-ray	1n0w	1.70 Å	B
	21–39	X-ray	3eu7	2.20 Å	A
MUTYH	76–362	X-ray	3n5n	2.30 Å	X/Y
	356–497	NMR	1x51	–	A

In the BRCA1 protein model (Modbase: a.a. 401–1458; template PDB 1w36, chain B; sequence identity 13%) we found only one 3D-cluster as listed in Table 4. Other two variants outside this cluster, the pathogenic germline variant p.S1298* is in close proximity to p.L1252fs but using a different 3D model (Modbase: a.a. 884–1786; template PDB 1jqn, chain A; sequence identity 11%) (diameter = 23.3 Å) (Fig. 7, panel b).

In the BRCA2 protein, we identified five 3D-clusters (see Table 4) in two 3D models from ModBase (a.a. 1895–2260; template PDB 4k6j, chain A; sequence identity 10%, and a.a. 2479–3191; template PDB 1mje, chain A; sequence identity 78%). Other variants in BRCA2 are also in close proximity but outside clusters: pathogenic germline p.2133_2134del and p.L2155fs (diameter = 15.5 Å); recurrent somatic p.T3085Nfs*26 and pathogenic germline p.K3083fs (diameter = 6.5 Å); and pathogenic germline p.D3095E, p.E3167* (diameter = 12.9 Å) (Fig. 7, panel c).

In the MUTYH protein, no 3D-clusters of somatic and germline variants were identified. However, according to the MUTYH crystal structure (PDB ID: 3n5n at 2.30 Å resolution; a.a. 76–362) and the solution NMR structure (PDB ID: 1x51; a.a. 356–497), pairs of variants are located in the same spatial region (Fig. 7, panel d).

In the MUTYH crystal structure (PDB ID: 3n5n at 2.30 Å resolution; a.a. 76–362) the pathogenic germline variant p.Y176C is located in the same spatial region as recurrent somatic variant p.S252T in PCa (diameter = 26.4 Å). The solution NMR structure (PDB ID: 1x51; a.a. 356–497) indicated that germline variant p.G393D is located in the same surface region as hotspot positions in different tumour types G393 and R423 (diameter = 24.5 Å) (see Fig. 7, panel d).

These findings suggest that the accumulation of variants in these spatial regions impairs protein interaction interfaces, and hence the biological function of the protein. The co-localization and 3D-clustering of germline and somatic variants onto the protein 3D-structure have also been applied by other authors to link rare predisposition variants to functional consequence^30^.

### Classification of DDR and non-DDR as cancer drivers across 33 tumour types

Here, we also compare the distribution of germline and somatic variants in 229 DDR and 1182 non-DDR components of the NetDDR network across mutation data in 33 different cancer types available from TCGA. First, we retrieved TCGA data in MAF format, generated by the MuTect2 workflow through the NCI Genomic Data Commons data portal (https://portal.gdc.cancer.gov/). Then, we applied the DiffMut method^31^, which compares the mutational profiles of genes across cancer genomes with their natural germline variation across healthy individuals. DiffMut^31^ uses the 1000 Genomes data as a background mutation rate. Data about DiffMut uEMD score and q-value for DDR and non-DDR is provided as Supplementary Files S3 and S4, respectively.

**Figure 8:**
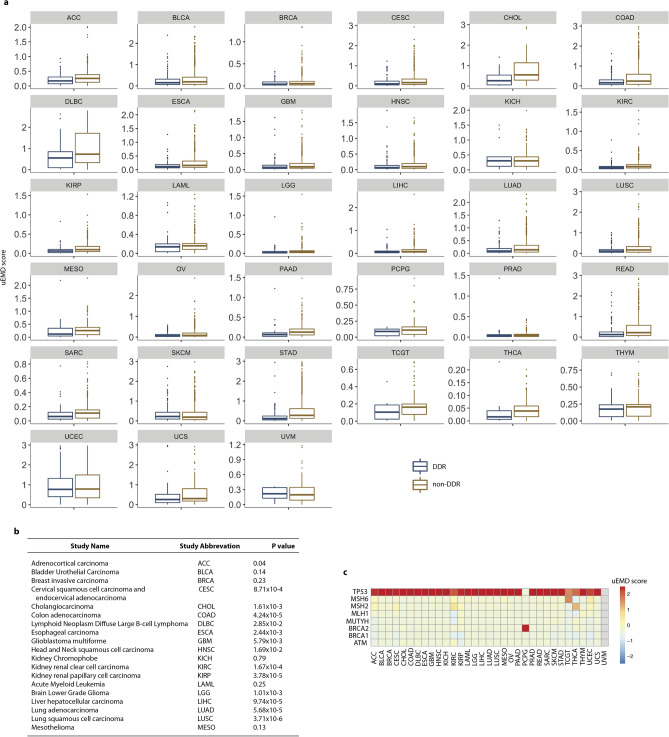
Study of DDR and non-DDR as cancer drivers across 33 tumour types. Panel (**a**) shows the DiffMut uEMD scores for the DDR and non-DDR genes across 33 tumour types studied in TCGA. BoxPlots indicate the 25th and 75th percentiles (box extent) and the median (centre line of each box). The whiskers extend from the hinge to the largest value no further than 1.5 × interquartile range from the hinge. Dots represent uEMD scores. The uEMD scores higher than 3 were excluded from the representation for clarity. Panel (**b**) shows P-values comparing DDR versus non-DDR genes based on the Wilcoxon rank-sum test. Panel (**c**) shows a heatmap of uEMD scores obtained by the different studies for the cancer-predisposition genes *ATM*, *BRCA1*, *BRCA2*, *MLH1*, *MSH2*, *MSH6, and TP53*.

Figure 8 shows the DiffMut results for DDR and non-DDR genes in 33 different cancer types. The non-DDR genes have significantly higher uEMD scores than DDR genes in most cancer types according to a Wilcoxon rank-sum test (Fig. 8, panels a and b). This could either mean that non-DDR genes have more cancer somatic mutations compared to the background rates of mutation in healthy individuals or that they have fewer background variants. As we said before, non-DDR genes accumulate fewer germline variants than DDR genes, which is in agreement with the DiffMut data.

We also show a heatmap with DiffMut uEMD scores for the cancer-predisposition genes *ATM*, *BRCA1*, *BRCA2*, *MLH1*, *MSH2*, and *MSH6* (Fig. 8, panel c). For comparison, we included the *TP53* gene that has an uEMD score greater than 1 in 25 out of 33 tumour types. It is important to mention that *TP53* was excluded from the study of the selected somatic variants identified in DDR genes extracted from COSMIC database (Supplementary Fig. S5), and their accumulation in different biological pathways (Fig. 3, panel c) due to the high number of variants, and for better visualization. Genes that score 0 are either those that were never observed to have a somatic mutation across the tumour samples or those that had higher background mutation rates than somatic mutation rates.

Overall, DiffMut data is in agreement with our previous results for DDR and non-DDR genes, and with the assumption that genic regions with a high ratio of rare variants to common ones are more intolerant to functional variation, so changes in these regions are more likely to be responsible for diseases.

## Discussion

DNA repair pathways protect cells against genomic damage; disruption of these pathways can contribute to the development of cancer. In this study we show that an integrative structural analysis of affected regions in the DDR protein-coding genes can help identify susceptibility to tumour development. Based on a combined analysis of the NetDDR interaction network and the curated list of germline and somatic variants mapped onto 1,411 nodes, we first observed that the percentage of DDR and non-DDR hits with annotations in COSMIC are very similar, while in ClinVar the percentage of DDR-hits with annotations is twofold higher than in non-DDR. This first observation suggested some bias in the ClinVar annotations towards the most studied genes reported in the literature, which may bias research findings or limit generalizability of the results.

A second observation from this analysis was the importance of the highly connected DDR genes associated with cancer-predisposition: *ATM*, *BRCA1*, *BRCA2*, *MLH1*, *MSH2*, and *MSH6*. These DDR genes are among the most affected in the COSMIC and ClinVar datasets, and their encoded proteins are central in the interaction network.

Proteins in the NetDDR network range in length from 44 to 7,968 amino acid residues, therefore we analysed the accumulation of germline and somatic variants as a function of protein length. We found that DDR hits accumulate a statistically higher number of germline variants per protein length than non-DDR (P-value = 5.0 × 10^–4^). Indeed, accumulation of variants is not uniform along the complete list of DDR proteins, showing a high number of germline variants accumulated in a few DDR hits. These DDR hits (BRCA1, BRCA2, MSH2, MLH1, and MSH6) coincide with highly connected and central proteins in the NetDDR interaction network. On the other hand, the numbers of somatic variants per protein length are similar in the DDR and non-DDR groups (P-value = 0.36). However, genes *ATM*, *ATRX*, *CHEK2*, *ERCC2*, *MLH1*, *MSH6*, and *SMARCA4* accumulate somatic variants above the 75th percentile of the distribution.

Our analysis of germline and somatic variants in the different pathways where DDRs are involved showed that the Homology Recombination, Fanconi Anemia, and Mismatch Repair pathways are the most affected by both types of mutations, whereas the Nucleotide Excision Repair pathway appears to be more affected by somatic variants in primary tumours than by germline pathogenic variants. The latter finding is in agreement with the results of other authors in that it shows an increased contribution of a somatic mutational pattern^25^.

Relatively few articles have investigated the structural and co-localization relationships between germline and somatic variants, and none have focused on a specific protein family^5^. Hence, in this article, we analysed different structural features: Pfam domains, 3D protein interfaces, protein flexible and/or intrinsically disordered regions (IDRs), and 3D clustering of variants. Interestingly, we discovered that 47.6% of somatic variants in metastatic tumours occur within Pfam domains, which is nearly double the value of 25% for germline variants. The percentage of somatic variants in metastatic tumours affecting 3D protein interfaces (36.4%) is threefold higher than the value of 11% to 13% in germline variants. Furthermore, it appears that accumulation of both germline and somatic variants within Pfam domains and 3D protein interfaces results in a synergistic effect that damages protein function.

In this article, as use cases in the DDR study, we investigated *ATM*, *BRCA1, BRCA2* and *MUTYH* genes, previously characterized in the mCRPC PROREPAIR-B cohort^14^. We analysed pathogenic germline variants identified in different cohorts of advanced PCa reported in the literature, recurrent somatic and germline variants in PCa, as well as hotspot positions in different tumour types collected from cBioPortal and the TCGA-PanCancer study of pathogenic germline variants in 10,389 adult cancers^28^. Using this large dataset, we observed that the vast majority of variants in ATM, BRCA1 and BRCA2 are located in flexible and/or IDRs, which are common in protein interaction interfaces. Moreover, it is possible to identify protein regions where germline pathogenic variants accumulate more than benign and VUS variants. These results together reinforce the hypothesis that there is a synergistic effect between germline and somatic variants affecting protein function and interactions^32^.

It is worth noting that a recent study into aggressive PCa, not limited to DDR genes, proposed 90 (out of 266, 34%) functionally related genes containing both germline and somatic variants^33^. The analysis used germline variants from genome-wide association studies (GWAS) and somatic variants identified in a cohort of 305 patients with aggressive tumours downloaded from The Cancer Genome Atlas (TCGA). Only 11 genes (i.e., *ATM*, *BRCA1*, *CCNH*, *CHEK2*, *FANCC*, *GADD45A*, *HERC2*, *NSMCE2*, *PARG*, *RAD23B*, *RAD51B*) are common to both our and their analyses. Note that *ATM* was not in the list of 266 genes with germline variants but in the differentially expressed genes associated with aggressive PCa and having somatic variants (see Supplementary Tables SA and S1A by Mamidi et al.^33^). These authors also identified different signalling pathways enriched for germline and somatic variants in which *ATM* is involved (i.e., PCa (P < 5.81 × 10^–6^), MSP-RON (P < 1.54 × 10^–5^), and P53 (P < 1.24 × 10^–4^)). Interestingly, these authors claim that genes containing germline variation did not have a high frequency of somatic variants, which is opposite to the findings we present here for the DDR genes. In our NetDDR network study, 118 (out of 229, 52%) protein-coding genes, including *ATM,* contain germline and somatic variants (see Supplementary File 1). The contradictory results are explained, in part, by the large number of non-DDR genes considered in the analysis done by Mamidi et al.^33^.

Co-localization and 3D-clustering of germline and somatic variants on the protein 3D-structure have previously been used to link rare predisposition variants to functional consequences^30^. Here we identified different clusters of variants within spheres with a 15-30 Å diameter that accommodate germline and somatic variants in ATM, BRCA1 and BRCA2, and propose that variants in the same 3D-cluster are part of a continuum of cancer-promoting changes, each with a relatively small but additive effect. The integrative structural analysis discussed in this article provides a comprehensive characterisation of affected regions in DDRs and can help in the understanding of an individual’s susceptibility to tumour development. One limitation of this approach is that some proteins may have incomplete, or no, structural coverage in the PDB, even when considering structures of homologous proteins, so no 3D cluster information can be obtained.

Overall, our findings indicated a synergistic effect between germline and somatic variants affecting protein domains and interactions in the DDR family genes. In particular, Pfam domains and protein interactions interfaces are more likely to be affected by somatic variants than pathogenic germline or VUS, suggesting that the emergence of a second somatic “hit” damages the protein’s function. On the other hand, we documented 3D clusters of pathogenic germline, recurrent somatic variants from primary and metastatic tumours, and hotspots positions in ATM and BRCA2. Proper structural characterization of germline and somatic variants is needed to better stratify cancer patients with affected driver genes.

## Materials and methods

### Protein–protein interaction network of DDRs

The aggregated protein–protein interaction (PPI) network for the 276 DDRs (DDR-hits) was constructed based on data from the BioGRID^19^, InWeb_IM^20^ and OmniPath^21^ databases. These databases were searched using the Metascape tool^22^. The initial PPI-Network generated in the first phase comprised 1,466 nodes (276 DDR-hits and 1,191 non-DDR-hits) and 38,494 edges. However, applying a filtering implementation from Metascape^22^ only 268 of the 276 DDR proteins have at least one interaction among themselves, and 229 of these 268 were considered to have enriched interactions or be “over-connected”. According to Metascape, an “over-connected” protein means that it has more than two interactions with other DDR-hits and possesses an over-connection p-value < 0.01^22^. In brief, Metascape produces an initial interaction map which is then pruned in a filtering step to detect. Later, for each connected component, the MCODE algorithm us iteratively applied to identify densely connected elements, excluding less connected proteins to identify the more relevant DDR hits, reduce false positives and prevent needles expansion and random associations. The final and more connected network, hereafter referred to as the ‘NetDDR’, contains 1,411 nodes (229 DDR-hits and 1,182 non-DDR-hits) and 36,522 edges. The NetworkAnalyzer plugin (http://apps.cytoscape.org/apps/networkanalyzer), was used to compute topology parameters for each node in the NetDDR undirected network (Supplementary Table S1).

### Functional enrichment and pathway analysis

A pathway and biological function enrichment analysis was done for the 268 over-connected proteins using the Metascape web tool (https://metascape.org). For the enrichment analysis we selected diverse ontologies such as: KEGG Functional Sets, Pathways, and Structural Complexes; GO Molecular Functions, Biological Processes, and Cellular Components, Oncogenic Signatures, Reactome Gene Sets, Canonical Pathways, BioCarta Gene Sets, CORUM and DisGeNET (Supplementary Fig. S1).

### Detection of densely connected regions in the NetDDR network

We used the MCODE algorithm, implemented in Metascape, to identify the highly interconnected proteins (or clusters) in the NetDDR network. The Metascape implementation of MCODE, can also make a GO enrichment analysis for each cluster identified by MCODE to assign “meanings” to the subnetwork or cluster components (Supplementary Fig. S2), where the top three best p-value terms were retained.

### A curated dataset of germline and somatic variants for the DDR genes and protein interactors in the NetDDR network

A curated dataset of somatic and germline variants was collected for the DDR genes involved in the NetDDR network, and also for their protein interactors (229 DDR-hits and 1,182 non-DDR-hits) from the COSMIC (https://cancer.sanger.ac.uk/cosmic) and ClinVar (https://www.ncbi.nlm.nih.gov/clinvar/) databases. Careful manual curation was required to compile a high-quality dataset of variants and to end up with an accurate set in each category (i.e. confirmed somatic, primary tumour or metastasis, confirmed germline, pathogenic, benign, unknown significance). After analysing the annotations, the selected variants, together with variants identified in different cohorts of mCRPC collected from the literature (https://pubmed.ncbi.nlm.nih.gov/) and TCGA (https://www.cancer.gov/tcga), were used to study the distributions across the protein sequences, Pfam domains, and protein 3D structures (see Supplementary File 1). Statistical significance tests for percentage comparisons following a successful Kruskal–Wallis test were done using the “N-1” Chi-squared test with Dunn correction for multiple comparisons.

#### Somatic variants in COSMIC

Cancer-associated somatic variants were selected from COSMIC v90^34^. This includes over 32,000 genomes, consisting of peer reviewed large-scale genome screening, and variants reported in The Cancer Genome Atlas (TCGA) and International Cancer Genome Consortium (ICGC). Only confirmed somatic variants were used when extracting variants associated with the selected DDR genes. Variants with undefined annotations (unclear syntax), as well as variants in non-coding regions, and synonymous variants were excluded. In order to focus on putative driver variants, only the variants identified in two or more tumour samples were considered for further analysis^35^. The pipeline described in this section is depicted in Supplementary Fig. S3.

#### Somatic and germline variants collected from the literature

Pathogenic germline variants identified in *ATM*, *BRCA1*, *BRCA2*, and *MUTYH* in the PROREPAIR-B Spanish cohorts and other mCRPC published cohorts (see Table 2 and Supplementary File 1), were collected for the analysis. In addition, a non-redundant dataset from 176 different studies containing 46,566 non-overlapping samples from 44,284 patients was explored using cBioPortal (https://www.cbioportal.org)^36,37^. Recurrent somatic and germline variants in PCa (n = 1,556) and hotspot positions in different tumour types (n = 44,284) in these genes are documented in Supplementary File 1.

#### Germline variants in ClinVar

Variants clearly classified as germline, according to *ClinVar* (Aug 2019), were annotated using VEP^38^ to check their genomic location and to include only the variants in protein coding regions. After discarding the variants with undefined or unclear annotation, we categorized the remaining variants into three different groups according to the Clinical Significance annotations provided by ClinVar. The groups were “pathogenic/likely pathogenic”, “benign/likely benign”, and “variants with unknown significance (VUS)”. For more details, see Supplementary Fig. S4.

#### Germline variants from TCGA

Germline variants classified as pathogenic or prioritized VUS were collected from TCGA^2^. We used *ATM*, *BRCA1*, *BRCA2*, and *MUTYH* as a proxy for the TCGA dataset (see Table 2 and Supplementary File 1).

### Structural modelling of proteins

Before mapping the germline and somatic variants onto the 3D-structures of PCa predisposition genes *ATM, BRCA1*, *BRCA2*, and *MUTYH*, we reviewed the RCSB PDB database (https://www.rcsb.org), which collects experimentally determined 3D-structures, and the ModBase repository (http://modbase.compbio.ucsf.edu) which generates homology-built protein 3D models (Tables 5 and 6). In the latter, the sequence identity between target proteins and those whose crystal structures were used as a template, ranged from 10 to 100% (Table 6).

**Table 6:** List of 3D models annotated in the ModBase database.

	Region	Template	Chain	Sequence identity (%)
ATM	1943–3055	4jsn	B	18
	1–917	2x19	B	13
	69–1030	3w3w	A	11
	2715–2939	4ajw	A	34
	1689–2272	2qmr	B	10
	606–1660	1u6g	C	12
	3024–3055	2kio	A	38
	698–1410	2qna	A	12
BRCA1	1646–1859	4igk	A	100
	884–1786	1jqn	A	11
	23–86	4qpl	A	44
	65–771	1xfx	A	14
	1680–1856	1l0b	A	66
	401–1458	1w36	B	13
BRCA2	1895–2260	4k6j	A	10
	2479–3191	1mje	A	78
	2848–3050	4tql	A	21
MUTYH	88–496	5dpk	A	35
	82–484	5dpk	A	38
	68–470	5dpk	A	38

Human ATM (3,056 a.a. UniProt: Q13315), BRCA1 (1,863 a.a. UniProt: P38398), BRCA2 (3,418 a.a. UniProt: P51587) and MUTYH (546 a.a. Unirpot: Q9UIF7) are multi-domain proteins whose complete crystallographic structure has not been determined yet. However, X-ray crystal structures (PDB id: 1jnx, 1n5o, 1t15, 1t29, 3eu7, 1n0w, 3n5n, 3n5n) and solution structures (PDB id: 6hka, 1jm7, 1oqa, 1x51, 5dpk) of different regions of these proteins are available. Furthermore, low-resolution electron microscopy models (PDB id: 5np0, 5np1) covering the complete sequence of ATM have been published. Additional data about the resolution, protein chains, and amino acid regions are shown in Table 5.

### Identification of affected protein functional domains, key residues, protein interaction interfaces, and 3D-clusters of germline and somatic variants

Protein domain annotations and flexible, or intrinsically disordered regions (IDRs), were extracted from the Pfam (https://pfam.xfam.org) and MobiDB (https://mobidb.bio.unipd.it) databases, respectively. Information on key functional residues (i.e. catalytic, ligand-binding, posttranslational modification) was collected from UniProt (https://www.uniprot.org) using the Structure-PPi system (https://rbbt.bsc.es/StructurePPI/Proteomics)^26^. Residues in protein interaction interfaces were identified via the Structure workflow implemented in the PCAWG-Scout online browser (http://pcawgscout.bsc.es)^39^. We also used the mutation3D program (http://mutation3d.org)^29^ to identify clusters of germline and somatic variants in the PDB and ModBase models. We ran mutation3D using the command recommended by its authors (./mutation3d < pdb_file >  < amino acid substitutions >  < CL-distance >  < protein length >  < number of bootstrapping iterations >) and parameters were set as follow: diameter or CL-distance = 30.0 Å, and number of bootstrapping iterations = 10,000.

### Comparison of germline and somatic mutational profiles across 33 different cancer types

We retrieved mutational data from TCGA in MAF format, generated by the MuTect2 workflow, through the NCI Genomic Data Commons data portal (https://portal.gdc.cancer.gov/). Then, we ran DiffMut (https://diffmut.princeton.edu/)^31^, with default parameters to compare the germline and somatic mutational profiles across 33 tumour types annotated in TCGA. The 1000 Genomes data was used as a background mutation rate. All TCGA mutations, with DiffMut uEMD score and q-value annotations, were filtered out for the 229 DDR and 1182 non-DDR genes. A Wilcoxon rank-sum test was used to assess statistical differences between germline and somatic mutational profiles in tumour samples.

## Supplementary Information

Supplementary Information 1.

Supplementary Information 2.

Supplementary Information 3.

Supplementary Information 4.

Supplementary Information 5.

## Data Availability

All data analysed during this study are included in this published article and its supporting information files.
