# Supplementary material for: A computational and structural analysis of germline and somatic variants affecting the DDR mechanism, and their impact on human diseases
Source: Sci Rep. 2021 Jul 12;11:14268. doi: 10.1038/s41598-021-93715-6 (PMC8275599; doi:10.1038/s41598-021-93715-6)
Supplement: Supplementary file 1 — Supplementary Information 1. [file 41598_2021_93715_MOESM1_ESM.docx]

Supplementary Table S1. **Topological parameters of the NetDDR network**

| **Parameters** | **1,411 nodes** | **229 DDR** |
| --- | --- | --- |
| AverageShortestPathLength | 2.36 | 2.2 |
| ClosenessCentrality | 0.43 | 0.46 |
| ClusteringCoefficient | 0.36 | 0.39 |
| Degree | 51.77 | 87.17 |
| Eccentricity | 3.7 | 3.52 |
| NeighborhoodConnectivity | 133.66 | 125.05 |
| Radiality | 0.73 | 0.76 |
| Stress | 70391.78 | 183985.76 |
| TopologicalCoefficient | 0.18 | 0.13 |

Supplementary Table S2. **Distribution of germline and somatic variants in Pfam domains and protein interfaces**

|  |  | **Total** | **No annotation** | **Inside**  **Pfam** | **Outside**  **Pfam** | **Inside Interfaces** | **Outside Interfaces** |
| --- | --- | --- | --- | --- | --- | --- | --- |
| **Germline**  **(ClinVar)** | **Pathogenic** | 10,301 | 532  (5.2%) | 2,522 (25.8%) | 7,247 (74.2%) | 1,268 (12.9%) | 8,501 (87.1%) |
|  | **Benign** | 1,117 | 123  (11%) | 137 (13.8%) | 857 (86.2%) | 72  (7.2%) | 922 (92.8%) |
|  | **VUS** | 28,248 | 3 737 (13.2%) | 6,136 (25%) | 18,375 (75%) | 2,788 (11.4%) | 21,723 (88.6%) |
| **Somatic**  **(COSMIC)** | **Metastasis** | 2,030 | 490  (24.1%) | 733 (47.6%) | 807 (52.4%) | 561 (36.4%) | 979 (63.6%) |
|  | **Primary** | 5,795 | 1,297  (22%) | 1,668 (37.1%) | 2,830 (62.9%) | 1,058 (23.5%) | 3,440 (76.5%) |


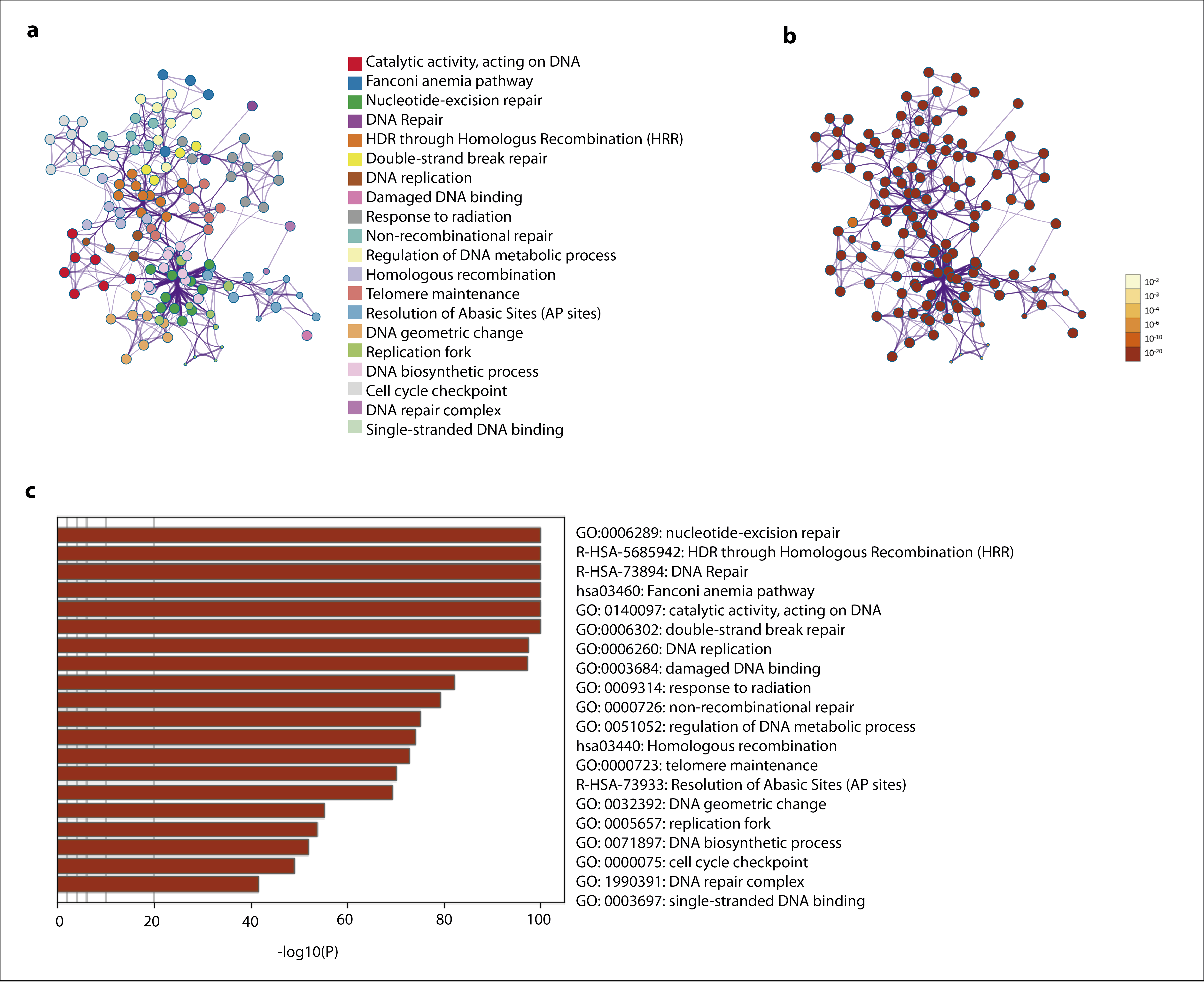


Supplementary Figure S1. **Functions and pathways enrichment analysis provided by Metascape.** The Network represents the top 20 clusters with their representative enriched terms—one per cluster. (a) Each node indicates an enriched term and is coloured by its cluster identity (i.e., nodes of the same colour belong to the same cluster). Node size is proportional to the number of input genes fall into that term. Terms with a similarity score > 0.3 are linked by an edge (the thickness of the edge represents the similarity score). One term from each cluster is selected to have its term description shown as label. (b) The same network but its nodes are colored by p-value, as shown in the legend. The dark the color, the more statistically significant the node is. (c) List of the top 20 statistically enriched terms. The terms can be GO/KEGG terms, canonical pathways, hall mark gene sets, etc.


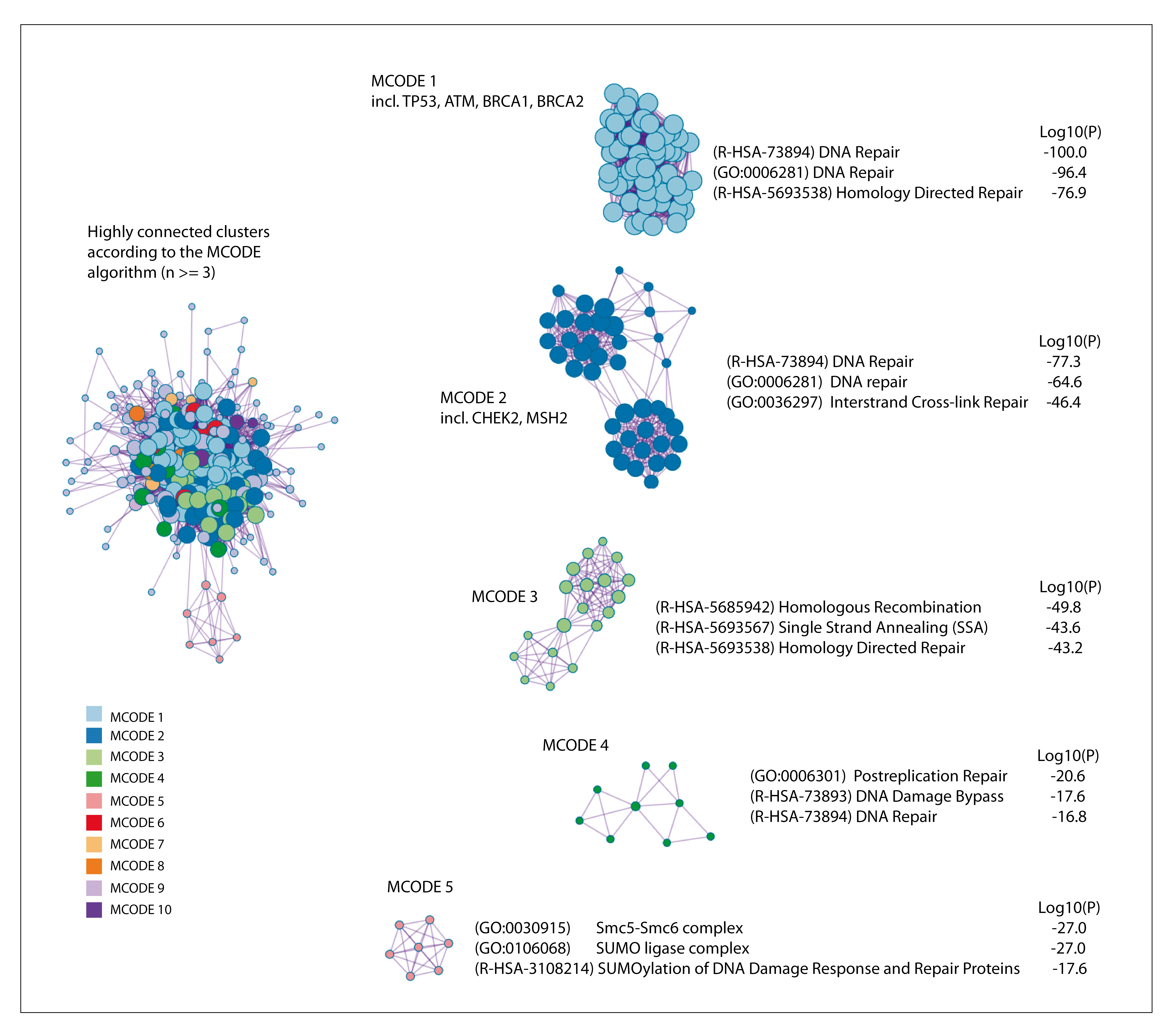


Supplementary Figure S2. **MCODE analysis of the NetDDR network as provided by Metascape**. Densely connected proteins (MCODE clusters) are highlighted. Each cluster is assigned a unique color. GO enrichment analysis was applied to each cluster to assign “meanings” to the network component, where top three best p-value terms were retained.

*
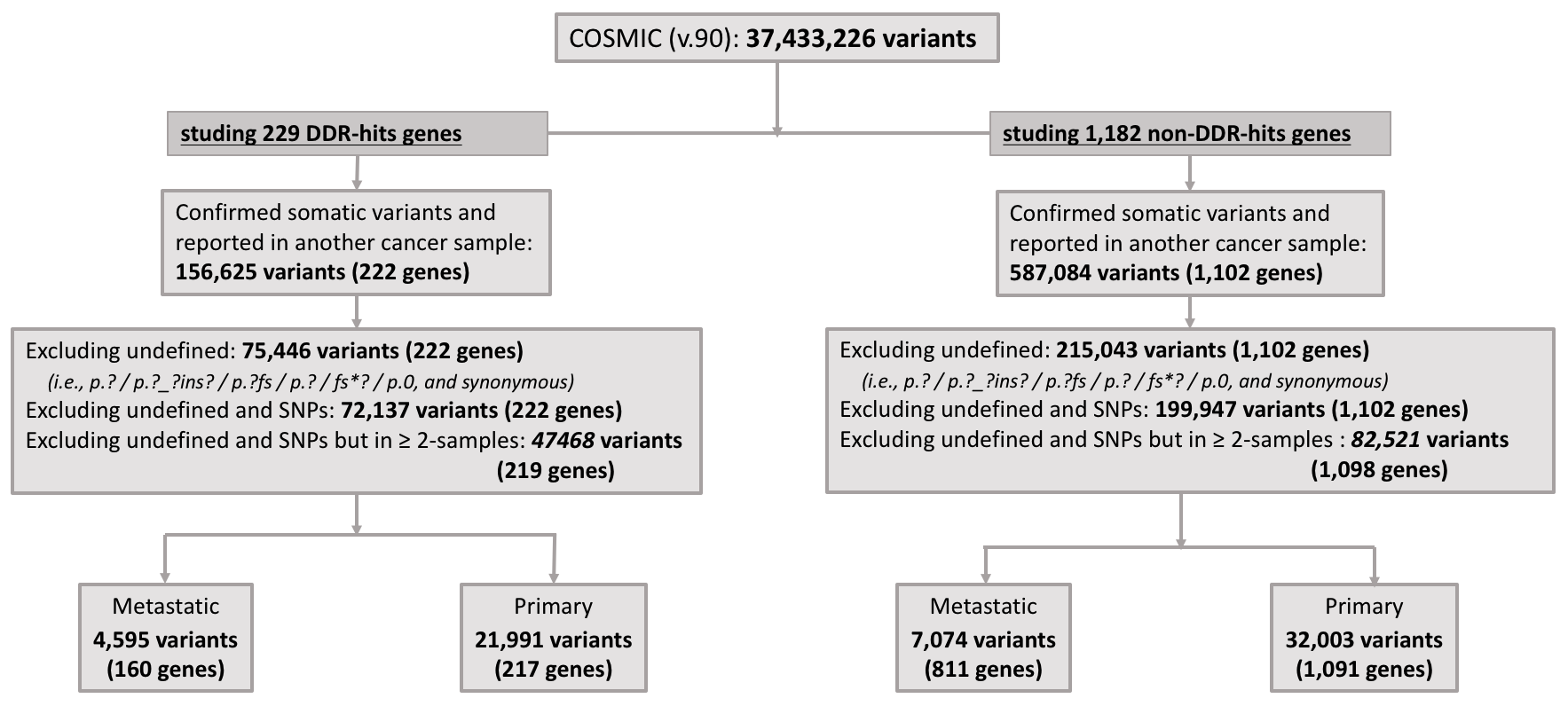
*

Supplementary Figure S3*.* **Flow chart for the classification of somatic variants extracted from COSMIC database**. DDR-hits and non-DDR-hits are according to the NetDDR network.

*
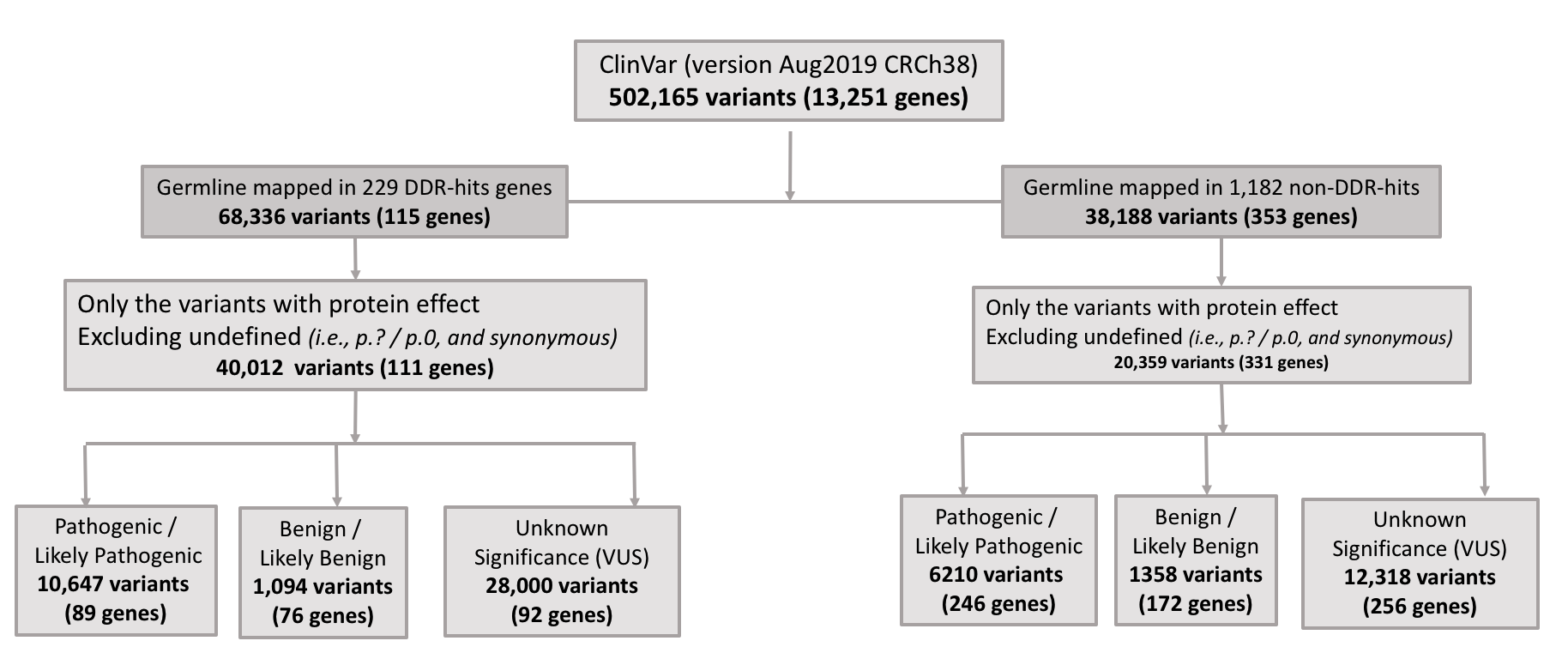
*

Supplementary Figure S4*.* **Flow chart for the classification of germline variants extracted from Clinvar database**. DDR-hits and non-DDR-hits are according to the NetDDR network.


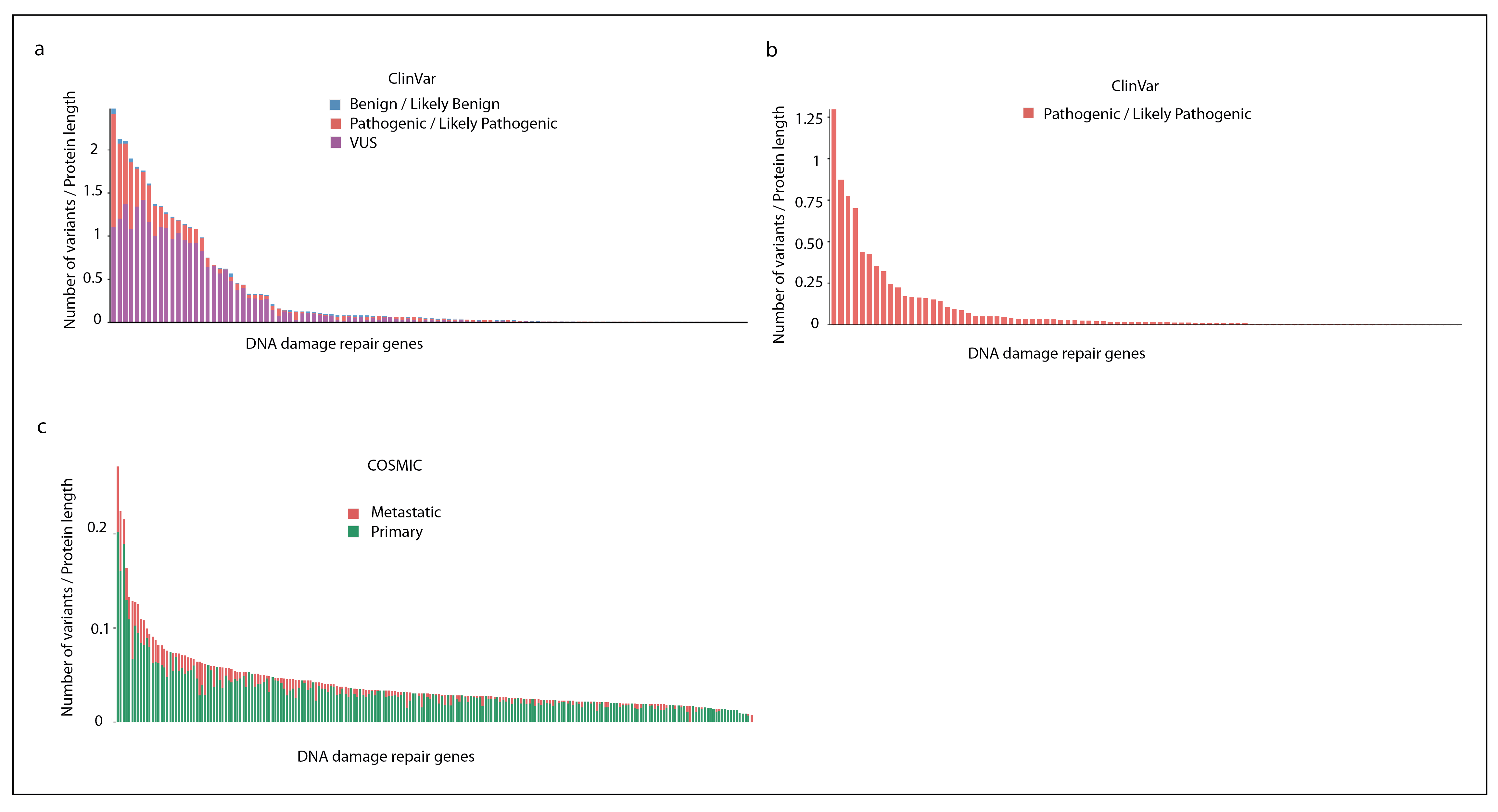


Supplementary Figure S5. **Summary of germline and somatic variants per protein length extracted from ClinVar and COSMIC databases in 229 DDR genes included in the NetDDR interaction network.** (a) Barplot of the selected germline variants identified in DDR genes and extracted from the ClinVar database. (b) Subset of pathogenic germline variants (only). (c) Barplot of the selected somatic variants identified in DDR genes and extracted from the COSMIC database. For a better visualization of the barplot, we exclude *TP53* that accumulate a large number of variants.


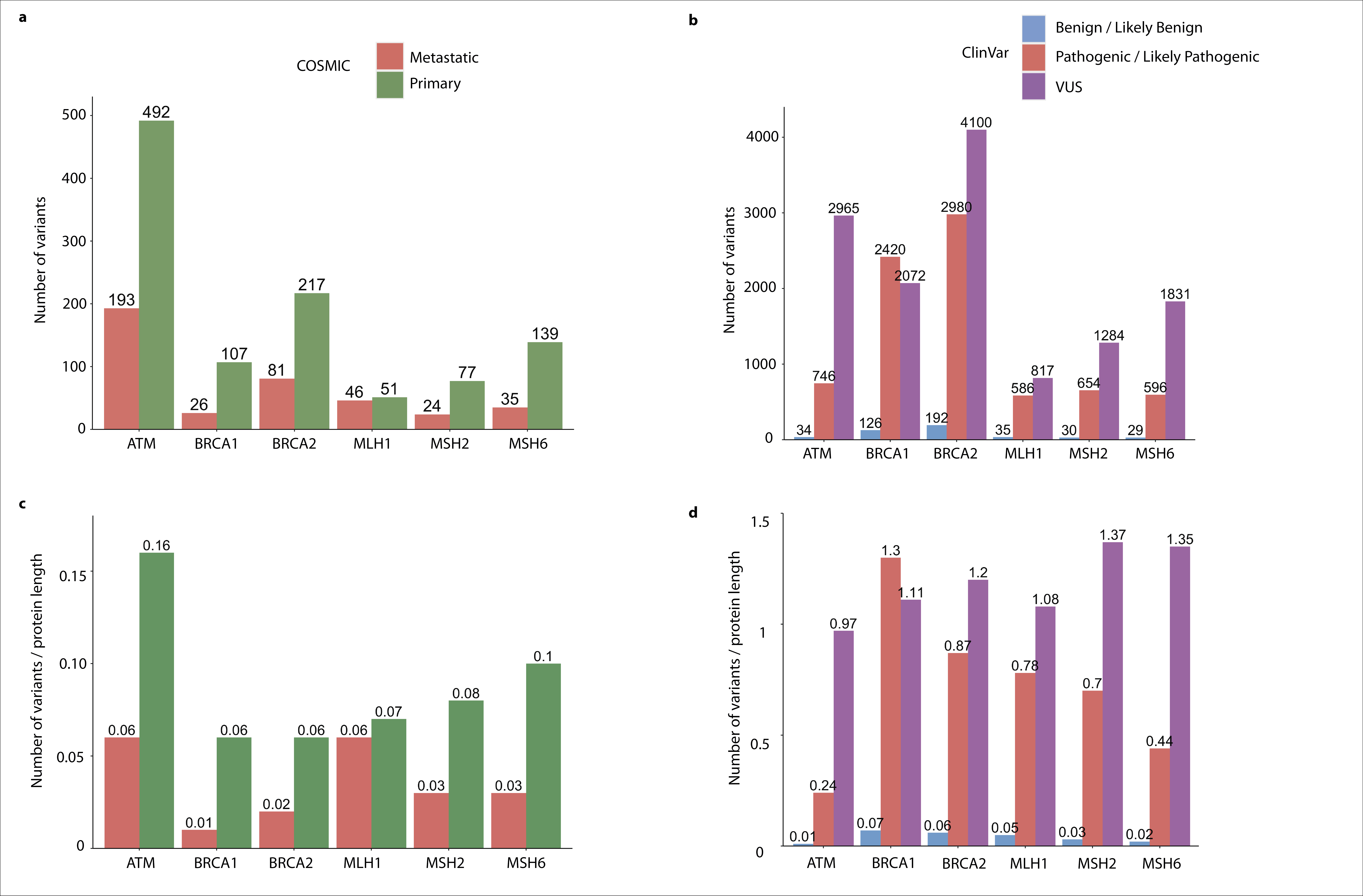


Supplementary Figure S6. **Number of variants affecting the most recurrent mutated genes in COSMIC and ClinVar databases**. (a) Barplot indicates the number of somatic variants affecting the selected genes according to COSMIC database. (b) Barplot indicates the number of germline variants affecting the selected genes according to ClinVar database. (c) Barplot indicates the number of somatic variants normalized per protein length affecting the selected genes according to COSMIC database. (D) Barplot indicates the number of germline variants normalized per protein length affecting the selected genes according to ClinVar database.

Supplementary_File 1 (MS Excel document). List of germline variants extracted from PubMed (pathogenic), TCGA (pathogenic and VUS), ClinVar (Pathogenic, Benign and VUS), and somatic variants from COSMIC (metastatic and primary) and cBioPortal (germline and recurrent somatic variants).

Supplementary_File 2 (MS Excel document). Functional annotations of germline and somatic variants using the Structure-PPI system.

Supplementary_File 3 (MS Excel document). DiffMut uEMD score, q-value and statistics for DDR.

Supplementary_File 4 (MS Excel document). DiffMut uEMD score, q-value and statistics for non-DDR.
